# Identification of colored wheat genotypes with suitable quality and yield traits in response to low nitrogen input

**DOI:** 10.1371/journal.pone.0229535

**Published:** 2020-04-21

**Authors:** Xiaoli Fan, Zhibin Xu, Fang Wang, Bo Feng, Qiang Zhou, Jun Cao, Guangsi Ji, Qin Yu, Xiaofeng Liu, Simin Liao, Tao Wang

**Affiliations:** 1 Chengdu Institute of Biology, Chinese Academy of Sciences, Chengdu, China; 2 Institute of Urban Agriculture, Chinese Academy of Agricultural Sciences, Chengdu, China; 3 University of Chinese Academy of Sciences, Beijing, China; 4 Innovative Academy for Seed Design, Chinese Academy of Sciences, Beijing, China; Institute of Genetics and Developmental Biology Chinese Academy of Sciences, CHINA

## Abstract

Colored wheat is a valuable resource that is rich in anthocyanins and minerals and thus contributes additional nutritional value to a healthy human diet. However, the effects of nitrogen fertilization on anthocyanin content (AC) and the balance between quality and yield still merit discussion. In this study, blue, purple, and common-colored wheat genotypes were used to investigate three nutrient quality traits, seven processing quality traits, three yield traits and seven grain morphology traits at three nitrogen levels in two years to excavate their possible plasticity under low-nitrogen stress and the tradeoffs among these traits. The highest AC was found in the blue genotypes followed by the purple genotypes. Analysis of variance (ANOVA) showed that AC could be significantly increased by reducing N application, especially in the purple genotypes. Therefore, growing colored wheat with low nitrogen input could allow efficient harvesting of grain with higher AC. However, the other nutrient quality traits and most processing quality traits were observed to decrease under low-nitrogen (LN) stress. Additionally, a correlation analysis indicated that the nutrient quality traits had stable tradeoffs with thousand kernel weight at all N levels because of the significantly negative correlations among them. Therefore, the additive main effect and multiplicative interaction (AMMI) model was used to further identify the most suitable colored genotypes with the best yield potential and also nutrient quality relative characteristics under LN stress. The blue lines Lanmai2999 and purple varieties Zhongkezinuomai 168 were found to be specifically adapted to LN stress with the highest AC values and showed stable performance in the other nutrient quality- and yield-related features. To further investigate the possible mechanism of anthocyanin accumulation in response to reduced N application, the expression of four genes (*TaCHS*, *TaFDR*, *TaCHI* and *TaANS*) involved in the anthocyanin synthesis pathway was evaluated. All four genes were downregulated under high nitrogen fertilizer application, indicating that anthocyanin synthesis in colored wheat might be inhibited by nitrogen fertilizer. Therefore, this research provided information for optimizing nitrogen fertilizer management in producing colored wheat and also demonstrated that it is efficient and economical to plant colored wheat genotypes in nitrogen-poor areas for use in a healthy human diet, improving the benefits of wheat planting and facilitating nitrogen pollution control.

## Introduction

Wheat (*Triticum aestivum* L.) is one of the most produced food crop on earth and plays an essential role in the human diet. Approximately 750 million tons are produced annually, together with maize and rice, forming the main staple food crop for 35% of the world’s population (FAO, 2018). Thus, quality and yield improvement are always the main targets of wheat breeders. In particular, traits related to processing and end-use quality, such as kernel hardness, test weight, water absorption, Zeleny sedimentation score, and wet gluten content, which directly or indirectly determine the commodity value of wheat, are the crucial optimization objectives in the wheat breeding process [1, 2].

Additionally, with the recent popularization of the concept of a healthy diet, in addition to food prices, which are mainly decided by production amounts and processing costs, people have become increasingly concerned about health and nutrition. Thus, the desire to “eat healthy” has become one of the most important factors influencing consumer food choices, and the nutrient quality traits in wheat, such as nutritive element content, have also gradually attracted breeders' attention [3, 4, 5]. Anthocyanin is a plant flavonoid pigment commonly found in traditional foods such as fruits, vegetables and grains, giving them their natural colors [6]. The primary ecophysiological function of anthocyanin is to attract seed dispersal agents and to protect plant tissues against abiotic and biotic stressors such as high radiation or pathogens [7]. Dietary intake of anthocyanins has profound health-promoting effects [8, 9], especially considering the plentiful evidence showing their contributions to human health as antioxidant, anti-inflammatory, antidiabetic, cardiovascular diseases, and anticancer agents, among other benefits [10]. Thus, food rich in natural anthocyanins is increasingly popular for a healthy diet.

Colored wheat, which is rich in anthocyanin content (AC) in the pericarp (purple wheat) or aleurone layer (blue wheat) compared with that of common-colored wheat (white or red wheat) [11, 12], can play a significant role in the prevention of various diseases associated with oxidative stress [13, 14, 15]. However, colored wheat germplasm resources, especially those with both optimal quality- and yield-related traits and availability for large-scale planting, are still lacking [5]. Thus, breeding colored wheat with both superior quality and superior yield features has become a popular objective to increase the value of processed wheat products.

In addition to genetic factors, abiotic environmental factors such as extreme photothermal characteristics, oxygen concentration and even fertilizer levels can affect anthocyanin metabolism [16]. For example, nitrogen (N) limitation can significantly promote pigmentation [17].

N is a major element critical for plant growth and affects both yield and quality formation[18]. In general, an increased N supply drives higher productivity and higher grain protein content [19], also improves some common processing and end-use relative features, such as wet gluten content, Zeleny sedimentation value, test weight and so on [20, 21, 22, 23]. However, excessive N fertilizer input can cause environmental pollution through N leaching and runoff, causing eutrophication of freshwater and marine ecosystems and N_2_O (a greenhouse gas) emission associated with denitrification by soil bacteria [19]. Thus, wheat with high tolerance to low N (LN) stress is an environmentally friendly germplasm resource.

However, colored wheat with a high commodity value that is adapted to LN stress and their corresponding N management still needs to be developed. Therefore, the present study quantified the genetic variation of 39 colored wheat germplasm lines with respect to nutrient quality traits, processing quality traits, grain morphology traits and yield conmponenets at different N levels, with the aim of (1) identifying colored wheat lines adapted to planting under LN stress; (2) investigating the effects of N input on grain color and other quality traits; and (3) evaluating the relationship between grain color and yield related traits at different N input levels.

## Materials and methods

### Plant materials and treatments

**Fig 1:**
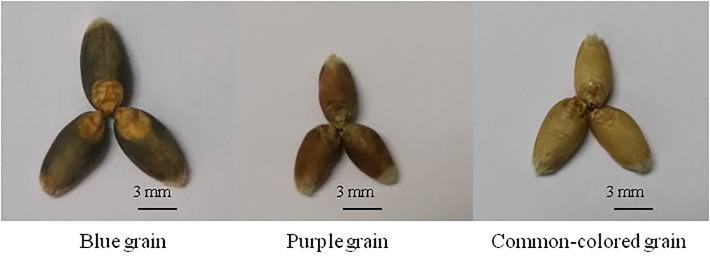
The color of three types of wheat grains.

Thirty-nine colored wheat lines (S1 Table), including color cultivars widely used in breeding program in China (such as Lanlimai and Luozzhen No.1), as well as varieties released in Southwest China in recent years (such as Chuanmai 42, Chuanyu 16, Mianmai 45 and Zhongkemai 138 and so on) and some intermediate lines bred by the Chengdu Institute of Biology, Chinese Academy of Sciences (CIBCAS), were selected as representatives of colored wheat: 10 blue, 22 purple and 7 common color (Fig 1).

The field experiments were conducted at Chengdu Plain Agricultural Ecology Test Station of Chinese Academy of Sciences, China (N 30°34′, E 103°52′) in 2016–2017 and 2017–2018. This test station is specifically provided to construct crop cultivation and breeding experiments by Chengdu Institute of Biology, Chinese Academy of Sciences. And we confirm that these field research in this study did not involve any endangered or protected planting species. Each year, soil samples were taken randomly at depths of 0–30 cm using a soil corer before sowing and submitted for analysis of available N (S2 Table). The wheat lines were planted in a randomized complete block using a split-plot design with three replications; the main plots consisted of three levels of N fertilizer application (low N, LN; medium N, MN; and high N, HN), and the subplot treatment was the 39 colored wheat cultivars. The subplot size was 2.0×1.0 m, containing 4 rows with 2 m long and 0.25 m apart. In the HN field, 120 kg N ha^-1^ was applied after sowing in both years. In MN, 90 kg N ha^-1^ was applied after sowing in both years. In the LN treatment, no nitrogen was applied during the entire growing period. Crop management followed local conventional agricultural practices, including disease and pest management, chemical weed control and other agronomic practices.

### Phenotypic evaluation and measurement

Three nutrient quality traits of the wheat grains, including AC, N concentration (NC) and protein content (GPC), were measured. Among them, the total AC was determined according to a previously published method [15]: a mixture of methanol acidified with 1 N HCl (85:15; v/v, 1 mL) was added to the samples (~100 mg) and then incubated at 4°C for 12 h in the dark. After centrifugation at 12 000 rpm for 10 min at 4°C, the supernatants were transferred into an ELISA plate. The absorbance of each sample was assessed at 510 nm using a Varioskan LUX Multimode Microplate Reader. A solution of methanol acidified with 1 N HCl was used as a blank. The total AC was corrected for dry matter and expressed as cyanidin 3-O-glucoside equivalent. The NC of the plant components was analyzed according to the following method: grains were collected at harvest and oven-dried to a constant weight at 60°C. The dried samples were first milled (<0.20 mm) using a mixer-grinder mill with mechanical modification for small-quantity samples for the measurement of N content [24]. The milled samples were then redried at 80°C for 48 h and weighed to 0.00001 g precision, encapsulated in tin capsules and analyzed for N%. The total N content was measured using an automatic N analyzer (BUCHI AutoKjeldahl Unit K-370; BUCHI Laboratory Equipment, Flawil, Switzerland). The protein content of the colored grains was determined according to the method of Hurkman and Tanaka [25].

Eight processing quality traits, including wet gluten content (WGC), Zeleny sedimentation value (ZEL), grain hardness (GH), test weight (TW), water absorption (WA), formation time (FT), dough stabilization time (DST) and maximum tensile resistance (MTR), were measured by near-infrared reflectance spectroscopy (NIRS) with a Perten DA-7250 instrument (Perten Instruments, Huddinge, Sweden) and expressed on the basis of 14% moisture. The measurements were calibrated using calibration samples according to the manufacturer’s instructions. Previous studies have confirmed that NIRS is a powerful method for measuring the quality traits of wheat; that it is rapid, effective, and convenient compared with traditional methods; and that it is appropriate for use in wheat breeding programs designed to improve quality-related traits [23].

Three yield components were investigated at maturity, including thousand-kernel weight (TKW), kernels per spike (KPS) and tiller number (TN). For each line in different replicates, six plants were randonmly chosen to measure TKW, KPS and TN. TKW were investigated using the Analysis System of WSeen SC-G Instrument (Zhejiang, China) by weighing more than 200 random kernels with two technical repeats; KPS were calculated from main and integrated tillers and TN were determined by the spike number harboring mature grains.

Seven grain morphology traits, including grain length (GL), grain width (GW), grain length/width ratio (LWR), grain diameter (GD), grain area (GA), grain perimeter (GP), and grain roundness (GR), were also measured by a WSeen SC-G Instrument analysis system (Zhejiang, China) with TKW evaluation.

### RNA extraction and qRT-PCR

Total RNA was isolated at 25 days after pollination from the grains of the purple wheat line Zhongkezinuomai168 (ZKZNM168), which is famous because of its high yield and was released by CIBCAS. TRIzol was used for RNA extraction in this study. After treatment with RNase-free DNase I (Promega), the total RNA (1 μg) was reverse transcribed using the TransScript II One-Step gDNA Removal and cDNA Synthesis SuperMix kits (TransGen Biotech). The reverse transcription products were used as templates for quantitative real-time PCR performed on a CFX96 Real-Time PCR system (Bio-Rad) using SYBR Premix Taq (TransGen Biotech) according to the manufacturer’s protocol. GAPDH was used as an internal control gene, and the relative expression levels were measured using the 2^-ΔΔCt^ analysis method. The primers used in RT-qPCR are listed in S3 Table.

### Statistical data analysis

One-way analysis of variance (ANOVA) was conducted to evaluate the variance, and Spearman’s rank correlation was performed to analyze phenotypic correlation coefficients between traits and significance between groups using SPSS 20.0 (SPSS, Chicago, IL, USA). The additive main effect and multiplicative interaction (AMMI) analysis was conducted by Genstat 19.0 (VSN International Ltd, UK).

## Results

### Correlation analysis at different N levels

The correlation analysis among three wheat nutrient quality traits, eight processing quality traits, three yield-related traits and seven grain morphology traits were evaluated under LN, MN and HN conditions (Table 1, S4 Table, S5 Table)

**Table 1:** Phenotypic correlations among the 21 investigated traits under low nitrogen treatment level.

	AC	NC	GPC	WGC	ZEL	GH	TW	WA	FT	DST	MTR	GA	GP	LWR	GL	GW	GD	GR	TKW	TN	KPS
AC	1.000																				
NC	0.681	1.000																			
GPC	0.573	0.943	1.000																		
WGC	0.541	0.907	0.977	1.000																	
ZEL	0.65	0.807	0.811	0.858	1.000																
GH	-0.011	-0.294	-0.3	-0.13	0.235	1.000															
TW	0.467	0.181	0.118	0.181	0.568	0.583	1.000														
WA	0.133	-0.093	-0.094	0.077	0.43	0.961	0.661	1.000													
FT	0.423	0.448	0.456	0.728	0.778	0.581	0.741	0.718	1.000												
DST	0.658	0.768	0.748	0.85	0.967	0.251	0.678	0.454	0.859	1.000											
MTR	0.345	0.198	0.103	0.221	0.462	0.571	0.642	0.333	0.789	0.598	1.000										
GA	-0.216	-0.338	-0.23	-0.102	-0.066	0.587	0.219	0.54	0.297	-0.052	0.181	1.000									
GP	0.234	0.069	0.111	0.246	0.391	0.653	0.549	0.713	0.681	0.441	0.55	0.796	1.000								
LWR	0.636	0.507	0.44	0.508	0.691	0.348	0.619	0.497	0.741	0.762	0.685	0.097	0.676	1.000							
GL	0.401	0.24	0.245	0.363	0.531	0.587	0.61	0.682	0.747	0.589	0.629	0.607	0.963	0.846	1.000						
GW	-0.661	-0.648	-0.52	-0.495	-0.613	0.104	-0.364	-0.051	-0.432	-0.67	-0.457	0.564	-0.042	-0.762	-0.303	1.000					
GD	-0.211	-0.326	-0.217	-0.092	-0.06	0.572	0.214	0.526	0.294	-0.047	0.172	0.999	0.792	0.089	0.602	0.571	1.000				
GR	-0.628	-0.487	-0.426	-0.5	-0.682	-0.371	-0.639	-0.52	-0.763	-0.763	-0.698	-0.151	-0.714	-0.993	-0.871	0.724	-0.144	1.000			
TKW	-0.494	-0.566	-0.463	-0.356	-0.375	0.443	-0.003	0.333	0.03	-0.355	-0.014	0.904	0.522	-0.239	0.289	0.784	0.906	0.187	1.000		
TN	-0.588	-0.381	-0.272	-0.273	-0.455	-0.107	-0.377	-0.226	-0.34	-0.5	-0.308	0.118	-0.237	-0.527	-0.358	0.524	0.113	0.525	0.309	1.000	
KPS	-0.08	-0.18	-0.217	-0.207	-0.11	0.216	0.104	0.116	-0.028	-0.126	0.06	0.088	0.078	0.058	0.08	0.006	0.074	-0.055	0.013	0.031	1.000

The numbers which were highlighted by blue color indicates significance at the level of 0.05.

The numbers which were highlighted by purple color indicates significance at the level of 0.01.

*AC* anthocyanin content, *NC* nitrogen concentration, *GPC* protein content, *WGC* wet gluten content, *ZEL* Zeleny sedimentation value, *GH* grain hardness, *TW* test weight, *WA* water absorption, *FT* formation time, *DST* dough stabilization time, *MTR* maximum tensile resistance, *GL* grain length, *GW* grain width, *LWR* grain length/width ratio, *GD* grain diameter, *GA* grain area, *GP* grain perimeter, *GR* grain roundness, *TKW* thousand-kernel weight, *KPS* kernels per spike, *TN* tiller number.

Overall, all three nutrient quality traits (AC, NC and GPC) were significantly positively correlated with each other, regardless of N treatment, indicating a possible common genetic background controlling these traits (Table 1, S4 Table, S5 Table).

Among the processing quality traits, only WGC, ZEL, FT and DST had significantly positive correlations with three nutrient quality traits (AC, NC and GPC), not affected by N treatment. However, apart from them, the significance of the correlation coefficients between the other processing quality traits and nutrient quality traits was not stable under the different N treatments. For example, TW was obviously correlated with AC under LN, with AC and NC under MN, and with all four nutrient traits under HN (Table 1, S4 Table, S5 Table).

For the relationships between the three yield-related traits and the nutrient traits, only TKW showed a consistently significant correlation with all four nutrient traits at all N levels, that is, negative relationships with AC, NC and GPC. These results indicated that grain weight components such as grain size and morphology traits might also be related to grain nutrient traits (Table 1, S4 Table, S5 Table). For the correlation of yield components with eight processing traits, only TKW-GH and TN-TW show stale significance under all N inputs, indicating the yield-quality tradeoffs were mainly existed between TKW and three nutrient traits.

To further specialize the contribution of the grain-related traits to these nutrient quality traits, seven grain size and shape traits were investigated. In detail, GW was significantly related to all three nutrient quality traits at all N levels, similar to TKW, showing negative relationships with AC, NC and GPC (Table 1, S4 Table, S5 Table). Thus, the relationship between TKW and nutrient traits might be mainly affected by GW. However, GL was always significantly positively related to AC at all three N levels, providing a possibility to simultaneously improve grain size and AC by finding their common genetic basis.

### Performance comparison of colored wheat lines

An ANOVA to analyze the different performance traits of blue, purple and common-colored wheat was conducted across all N levels. In total, the three kinds of wheat with different colored grains could be distinguished in regard to most grain features (Table 2).

**Table 2:** Comparison of 21 investigated traits of different colored wheat lines and of wheat lines under different N levels.

Traits	For different color			For different N levels		
	Blue wheat	Purple wheat	Common-colored wheat	LN	MN	HN
AC (μg/g)	68.54±3.80 a	25.52±1.37 b	8.65±0.45 c	38.23±3.03 a	36.32±4.46 a	26.34±4.34 b
NC (μg/g)	25.90±0.64 a	22.92±0.42 b	20.93±0.53 c	20.00±0.30 a	22.93±0.36 b	27.05±0.40 c
GPC (%)	16.35±0.33 a	15.06±0.20 b	13.47±0.32 c	13.50±0.21 a	14.87±0.19 b	16.95±0.19 c
WGC (%)	33.81±0.71 a	30.90±0.44cb	28.34±0.77 c	27.62±0.40 a	30.64±0.39 b	35.29±0.43 c
ZEL (ml)	46.85±1.40 a	39.90±0.68 b	34.38±1.60 c	34.29±0.95 a	40.75±0.91 a	47.03±0.84 b
GH	63.05±1.29 a	59.83±0.77 c	66.08±1.05 b	59.13±1.33 a	62.95±0.84 b	63.27±0.86 b
TW (g/L)	774.79±1.91 a	759.68±1.05 b	765.82±1.95 b	763.70±1.92	762.57±1.94	764.31±1.77
WA (%)	61.45±0.78 a	57.48±0.49 b	62.71±0.62 a	58.99±0.91	59.55±0.57	59.77±0.64
FT (min)	3.21±0.16 a	1.88±0.10 b	2.34±0.22 b	1.52±0.12 a	2.24±0.12 b	3.16±0.14 c
DST (min)	16.70±0.99 a	10.81±0.46 b	8.70±1.01 b	7.77±0.62 a	11.84±0.67 b	16.21±0.61 c
MTR (B.U.)	660.66±20.86 a	493.86±9.20 b	639.97±25.89 a	572.94±13.31	577.47±19.39	568.15±24.54
GA (mm^2^)	15.95±0.24 a	16.15±0.11 a	16.84±0.15 b	16.45±0.16 a	16.35±0.15 ab	15.88± 0.18 b
GP (mm)	16.78±0.13 a	15.97±0.06 b	16.66±0.09 a	16.46±0.11	16.31±0.10	16.14±0.11
LWR	2.34±0.03 a	1.93±0.01 b	2.08±0.02 c	2.09±0.03	2.07±0.03	2.03±0.03
GL (mm)	6.83±0.06 a	6.19±0.03 b	6.57±0.04 c	6.51±0.06	6.40±0.06	6.36±0.06
GW (mm)	2.96±0.03 a	3.24±0.01 b	3.21±0.01 b	3.20±0.02	3.16±0.03	3.12±0.03
GD (mm)	4.47±0.03 a	4.50±0.02 a	4.59±0.02 b	4.54±0.02	4.53±0.02	4.46±0.02
GR	0.44±0.01 a	0.53±0.01 b	0.49±0.01 c	0.51±0.01	0.49±0.01	0.50±0.01
TKW (g)	42.32±0.99 a	45.99±0.41 b	48.71±0.66 c	46.50±0.69	45.90±0.63	44.20±0.78
TN	5.85±0.33 a	7.69±0.36 b	6.96±0.40 ab	5.03±0.11 a	7.48±0.30 b	8.75±0.49 c
KPS	55.93±1.83	56.99±0.95	57.03±1.37	56.63±1.19	56.71±1.03	56.83±1.63

Different letters followed the numbers represent the significant difference among different colored wheat lines or different N levels, *P*<0.05.

*AC* anthocyanin content, *NC* nitrogen concentration, *GPC* protein content, *WGC* wet gluten content, *ZEL* Zeleny sedimentation value, *GH* grain hardness, *TW* test weight, *WA* water absorption, *FT* formation time, *DST* dough stabilization time, *MTR* maximum tensile resistance, *GL* grain length, *GW* grain width, *LWR* grain length/width ratio, *GD* grain diameter, *GA* grain area, *GP* grain perimeter, *GR* grain roundness, *TKW* thousand-kernel weight, *KPS* kernels per spike, *TN* tiller number, *LN* low nitrogen treatment, *MN* medium nitrogen treatment, *HN* high nitrogen treatment.

In detail, for the nutrient quality traits, AC, NC and GPC were all higher in blue wheat than in the other wheat colors. In addition, purple wheat also had obviously higher AC, NC and GPC than common-colored wheat (Table 2).

For the processing quality traits, blue wheat also showed significantly higher values than purple and common-colored wheat for the measured processing quality traits, except for GH (Table 2).

For grain size/shape, it was not easy to distinguish colored wheat from common-colored wheat based on only their similar grain size parameters, such as GA and GP. However, blue wheat had the longest grains, and purple wheat had the shortest grains, and overall, the grain shapes were obviously different among blue, purple and common-colored wheat, that is, blue wheat had the largest LWR and purple wheat had the roundest grains (GR) (Table 2).

For the three yield components, only TKW showed differences among the three kinds of wheat, with common-colored wheat > purple wheat > blue wheat, possibly due to differences in their grain shapes. However, their KPS were nearly the same (Table 2).

### Effects of different nitrogen fertilizer treatments on colored wheat lines

Overall, nitrogen application remarkably affected wheat nutrient quality (Table 2). Under LN stress, AC was higher than that in MN and HN conditions. However, NC and GPC were positively related to N fertilizer application levels, being highest under HN and lowest under LN. Among the processing quality traits, WGC, ZEL, GH, FT and DST were also sensitive to LN stress and showed increasing values with increasing N application. However, for grain size/shape traits and yield-related traits, only GA and TN were significantly affected by different N levels. These results indicated that quality traits, especially nutrient quality traits, might have more plasticity with respect to N levels than do grain weight and morphology traits, which have been proven to have higher heritability. And thus it provided an idea to select sepcialized and optimized N managements to improve quality of wheat with stably superior grain weight perfomance, with the aim to balance yield potential and quality characteristics.

Based on the results of investigating these three different colored wheat groups, all showed trends similar to the overall trends for all the nutrient quality traits and most of the other traits (Table 3). It is interesting that the AC in blue and purple wheat did not significantly decrease when N application was increased beyond MN, while their NC and GPC values both obviously increased, indicating that in colored wheat, the MN level might be enough to synthesize not only AC but also NC and GPC, leading to more economical and efficient N use.

**Table 3:** Comparison of 21 investigated traits among different colored wheat lines under different N levels.

Traits	Blue wheat	Purple wheat	Common-colored wheat						
	LN	MN	HN	LN	MN	HN	LN	MN	HN
AC (μg/g)	78.23±5.74 a	74.10±7.49 a	53.30±3.29 b	28.75±2.65 a	27.56±2.07 a	20.25±2.03 b	9.92±0.55 a	8.85±0.28 a	7.16±0.48 b
NC (μg/g)	22.46±0.40 a	25.23±0.56 b	29.99±0.44 c	19.42±0.25 a	22.53±0.37 b	26.81±0.32 c	18.30±0.35 a	20.88±0.49 b	23.61±0.35 c
GPC (%)	14.83±0.28 a	16.07±0.34 b	18.06±0.31 c	13.79±0.10 a	14.78±0.14 b	16.71±0.18 c	11.99±0.24 a	13.08±0.21 b	15.19±0.21 c
WGC (%)	30.08±0.71 a	33.23±0.60 b	38.11±0.59 c	27.46±0.37 a	30.30±0.38 b	34.93±0.43 c	24.61±0.50 a	27.99±0.44 b	32.41±0.57 c
ZEL (ml)	40.28±1.89 a	46.59±1.85 b	53.67±1.35 b	34.13±0.60 a	40.29±0.68 b	45.29±0.73 c	26.23±0.82 a	33.86±0.85 b	43.04±0.80 c
GH	61.20±2.29	65.14±2.29	62.83±2.19	55.11±1.44 a	62.17±0.95 b	62.22±1.01 b	68.79±1.87 a	62.25±1.49 b	67.19±1.23 ab
TW (g/L)	774.56±2.79	774.65±3.77	775.16±3.66	757.60±1.97	756.57±1.89	758.87±1.64	767.38±4.50	764.16±2.98	765.91±2.83
WA (%)	62.47±1.45	61.02±1.34	60.85±1.33	56.40±1.02	57.69±0.66	58.36±0.83	65.28±0.89 a	60.19±0.77 b	62.66±0.58 ab
FT (min)	2.44±0.24 a	3.05±0.21 a	4.14±0.17 b	1.18±0.09 a	1.86±0.11 b	2.61±1.13 c	1.27±0.08 a	2.28±0.23 b	3.46±0.17 c
DST (min)	12.25±1.38 a	16.47±1.42 a	21.37±0.97 b	7.01±0.41 a	10.96±0.53 b	14.47±0.47 c	3.77±0.33 a	8.01±0.70 b	14.33±0.44 c
MTR (B.U.)	582.32±20.20 a	660.53±40.84 ab	739.13±27.62 b	490.70±11.33	521.15±18.25	469.73±16.12	624.17±33.03	610.55±50.67	685.18±50.16
GA (mm^2^)	16.31±0.37	16.06±0.41	15.47±0.48	16.26±0.17	16.31±0.17	15.89±0.21	17.24±0.31	16.87±0.24	16.41±0.17
GP (mm)	17.01±0.21	16.76±0.22	16.60±0.26	16.07±0.11	16.01±0.10	15.84±0.11	16.90±0.16	16.61±0.15	16.47±0.12
LWR	2.37±0.03	2.30±0.08	2.36±0.06	1.96±0.04 a	1.90±0.01 b	1.93±0.02 ab	2.10±0.02	2.04±0.33	2.09±0.03
GL (mm)	6.94±0.10	6.80±0.10	6.75±0.11	6.26±0.05	6.18±0.05	6.14±0.05	6.69±0.07	6.52±0.08	6.49±0.07
GW (mm)	2.98±0.04	2.99±0.05	2.90±0.07	3.23±0.02	3.28±0.02	3.22±0.02	3.23±0.03	3.24±0.03	3.16±0.02
GD (mm)	4.52±0.05	4.50±0.06	4.41±0.07	4.51±0.02	4.53±0.02	4.47±0.30	4.64±0.04	4.61±0.03	4.54±0.02
GR	0.43±0.01	0.44±0.01	0.43±0.01	0.52±0.003 a	0.54±0.003 b	0.53±0.004 ab	0.49±0.01	0.50±0.01	0.49±0.01
TKW (g)	43.48±1.43	43.06±1.64	40.48±2.05	46.52±0.71	46.35±0.63	45.08±0.79	50.76±1.25 a	48.54±0.93 ab	46.82±0.77 b
TN	4.42±0.22 a	6.48±0.54 b	6.65±0.63 b	5.28±0.13 a	7.98±0.42 b	9.81±0.71 c	5.10±0.09 a	7.33±0.48 b	8.45±0.65 b
KPS	55.58±3.25	56.30±2.43	55.92±3.98	56.68±1.34	56.45±1.21	57.83±2.22	57.98±2.62	58.14±2.86	54.97±1.67

Different letters followed the numbers represent the significant difference among three nitrogen fertilizer levels, *P*<0.05.

*AC* anthocyanin content, *NC* nitrogen concentration, *GPC* protein content, *WGC* wet gluten content, *ZEL* Zeleny sedimentation value, *GH* grain hardness, *TW* test weight, *WA* water absorption, *FT* formation time, *DST* dough stabilization time, *MTR* maximum tensile resistance, *GL* grain length, *GW* grain width, *LWR* grain length/width ratio, *GD* grain diameter, *GA* grain area, *GP* grain perimeter, *GR* grain roundness, *TKW* thousand-kernel weight, *KPS* kernels per spike, *TN* tiller number, *LN* low nitrogen treatment, *MN* medium nitrogen treatment, *HN* high nitrogen treatment.

To further investigate the tolerance of different colored wheat lines to low N stress to futher facilitate N-input restriction and reduce N pollution, the amplitude of the variation was calculated as follows: the difference between each measured value from LN and HN was estimated, and these differences were used to obtain the percentage of each original measured value based on the HN condition, defined as (LN-HN)/HN×100%. The results showed that AC in purple wheat was more sensitive to LN stress (increased 58.18%) and accumulated more under LN conditions than blue wheat (increased 47.11%), although its GPC also decreased slightly but not significantly (Table 4).

**Table 4:** Performance variation proportion under LN stress of those under HN conditions.

Traits	Blue wheat (%)	Purple wheat (%)	Common-colored wheat (%)
AC (μg/g)	47.11±6.70	58.18±8.85	38.54±10.64
NC (μg/g)	-25.12±0.91	-27.46±0.88	-22.44±1.71
GPC (%)	-19.89±1.41	-20.17±0.76	-21.32±1.28
WGC (%)	-21.07±0.74	-21.31±0.74	-24.01±1.61
ZEL (ml)	-25.18±1.94 a	-24.41±1.94 a	-38.91±3.05 b
GH	-2.67±1.07 a	-11.65±1.05 b	2.38±2.03 c
TW (g/L)	-0.07±0.32	-0.17±0.14	0.19±0.36
WA (%)	2.64±0.45 a	-3.45±0.56 b	4.18±0.97 a
FT (min)	38.91±5.65	47.560±8.99	58.52±12.30
DST (min)	34.54±7.03 a	38.05±9.03 a	85.39±13.23 b
MTR (B.U.)	13.82±4.23 a	-8.35±3.54 b	13.25±3.76 a
GA (mm^2^)	5.70±1.57	2.45±0.84	5.00±1.16
GP (mm)	2.72±0.46	1.48±0.37	2.58±0.29
LWR	0.38±1.51	1.58±0.38	0.85±1.00
GL (mm)	2.96±0.44	2.03±0.35	2.93±0.17
GW (mm)	2.91±1.41	0.54±0.52	2.25±0.98
GD (mm)	2.67±0.79	1.03±0.41	2.31±0.58
GR	0.23±1.45	-1.25±0.37	-0.62±0.86
TKW (g)	8.68±3.42	3.47±1.34	8.49±2.48
TN	-30.53±4.29	-42.00±3.54	-37.16±5.47
KPS	1.54±5.62	0.39±3.57	5.65±4.18

Different letters followed the numbers represent the significant difference among variation proportion, P<0.05.

*AC* anthocyanin content, *NC* nitrogen concentration, *GPC* protein content, *WGC* wet gluten content, *ZEL* Zeleny sedimentation value, *GH* grain hardness, *TW* test weight, *WA* water absorption, *FT* formation time, *DST* dough stabilization time, *MTR* maximum tensile resistance, *GL* grain length, *GW* grain width, *LWR* grain length/width ratio, *GD* grain diameter, *GA* grain area, *GP* grain perimeter, *GR* grain roundness, *TKW* thousand-kernel weight, *KPS* kernels per spike, *TN* tiller number, *LN* low nitrogen treatment, *HN* high nitrogen treatment.

### Identification of colored wheat lines suitable for LN stress

As shown in Tables 2 and 3, AC appears to accumulate when N application is reduced; therefore, colored wheat, is more suitable for planting under LN conditions to maximize its inherently high AC. To further identify specific genotypes adapted to LN stress with stable quality and yield potential, the AMMI model was used to evaluate the stability and adaptation of the blue and purple wheat lines for different N applications.

**Fig 2:**
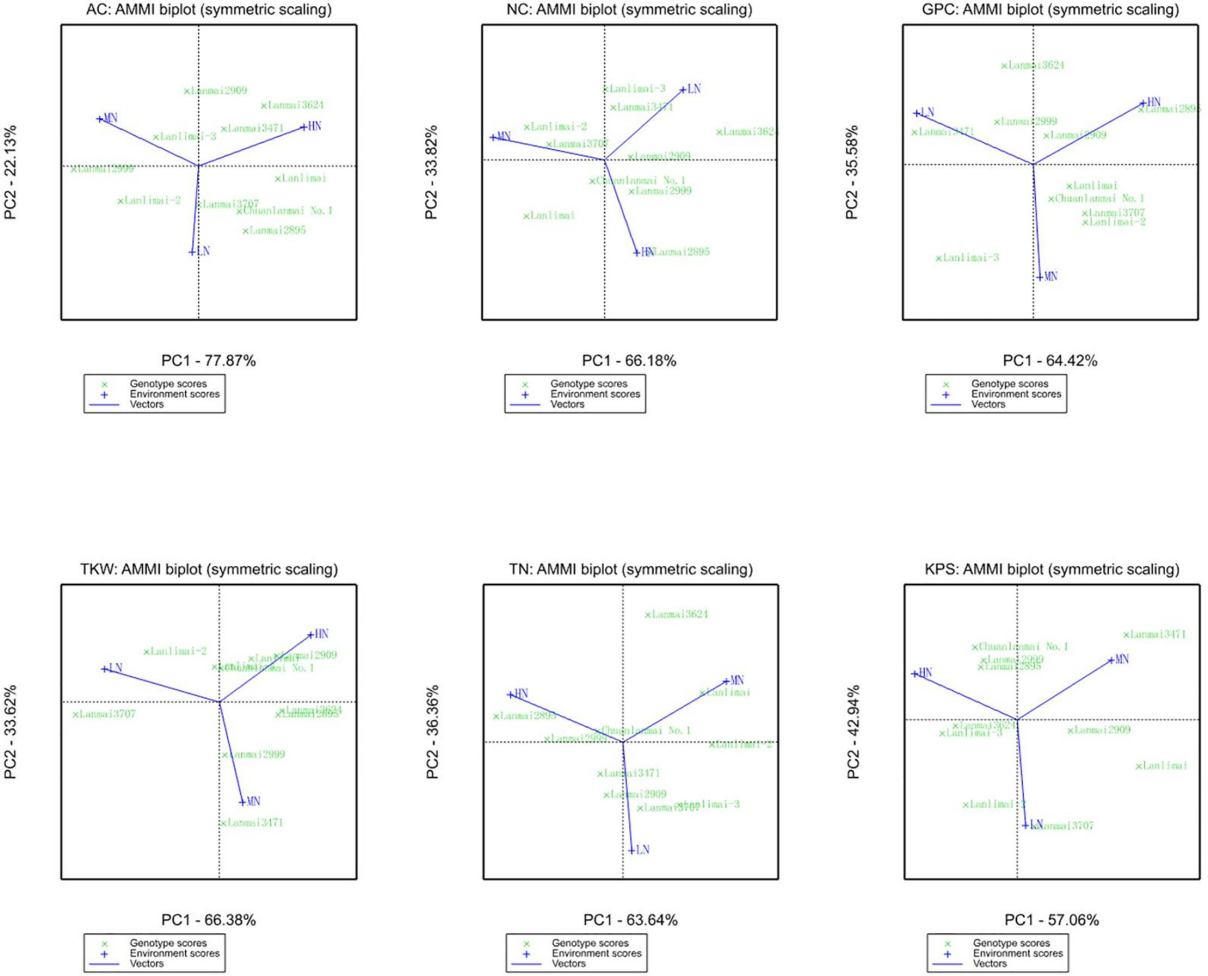
AMMI analysis of blue wheat lines for nutrient quality and yield composition traits.

For the AC in blue wheat genotypes (Fig 2, Table 5), the lowest AMMI stability values (ASVs) were observed for Lanmai3707, Lanmai2909 and Lanmai3471, which indicated a higher stability of these genotypes under all N treatments; however, their AC values were relatively low. Higher AC values were observed in Lanmai2999, Lanlimai-3 and Lanlimai-2, in decreasing order. The highest ASV belonged to Lanmai2999, followed by Lanlimai and Lanlimai-2, indicating that these genotypes were more specifically adapted to certain N levels. Fig 2 depicts genotype-environment affinity as orthogonal projections of the genotypes on the environmental vectors to identify the best genotypes with respect to N levels. Lanmai2999 and Lanlimai-2 were better adapted to both LN and MN than the other genotypes, and they also had higher AC values.

**Table 5:** Average and AMMI stability value (ASV) of nutrient quality traits and yield composition traits for the blue wheat lines.

Genotypes	Nutrient quality traits						Yield composition traits					
	AC (μg/g)		NC (μg/g)		GPC (%)		TKW (g)		TN		KPS	
	Mean	ASV	Mean	ASV	Mean	ASV			Mean	ASV	Mean	ASV	Mean	ASV
Chuanlanmai No.1	78.23	4.49	23.59	0.30	14.74	0.30	45.00	0.49	5.51	0.43	63.99	1.95
Lanmai2895	53.30	5.31	25.87	1.20	16.48	1.27	47.77	1.69	6.89	2.09	62.25	1.52
Lanmai2909	68.72	2.56	25.24	0.48	16.36	0.23	48.98	1.76	4.94	0.56	58.46	1.51
Lanmai2999	98.39	13.16	26.28	0.58	16.59	0.49	32.74	0.78	6.27	1.25	65.30	1.58
Lanmai3471	51.81	2.88	27.16	0.52	17.33	1.36	41.10	1.76	5.16	0.48	39.82	3.56
Lanmai3624	52.06	7.09	24.45	2.13	15.29	0.70	47.79	1.78	8.91	1.26	52.25	1.74
Lanmai3707	60.11	1.16	25.64	1.03	16.14	0.66	38.94	4.10	5.63	0.68	58.70	2.32
Lanlimai	55.42	8.36	28.34	1.53	17.75	0.43	39.91	1.10	5.81	1.38	46.30	3.57
Lanlimai-2	81.21	8.29	25.60	1.46	15.99	0.69	38.47	2.20	5.06	1.46	64.67	2.32
Lanlimai-3	86.17	4.57	26.71	0.66	16.82	1.22	42.52	0.53	4.30	1.10	47.59	2.13

The highlighted color is deepening with the corresponding average values or ASVs increasing

*AC* anthocyanin content, *NC* nitrogen concentration, *GPC* protein content, *TKW* thousand-kernel weight, *KPS* kernels per spike, *TN* tiller number.

The other two nutrient quality traits (NC and GPC) were positively correlated with AC, while three yield components (TKW, TN and KPS) were negatively correlated with AC. All five traits were generally depressed by reducing N application. Thus, their stability and adaptation to LN stress were further considered to identify the most suitable genotypes with not only high AC but also satisfactory quality and yield features.

Between Lanmai2999 and Lanlimai-2, which were adapted to LN stress with high AC, Lanmai2999 showed lower ASVs for NC, GPC, TKW, TN and KPS than did Lanlimai-2, indicating that these traits in Lanmai2999 are more stable under all N levels (Table 5). Similarly, the other processing traits are also more stable in Lanmai2999, compared with Lanlimai-2 (S6 Table, S1 Fig). On the other hand, Lanmai2999 had higher NC, GPC, TN and KPS (with the highest KPS value overall) than Lanlimai-2 but had lower TKW and GH. Thus, Lanmai2999 might be the most suitable blue soft genotype to be planted under LN stress with high AC; however, its TKW should be further improved.

**Fig 3:**
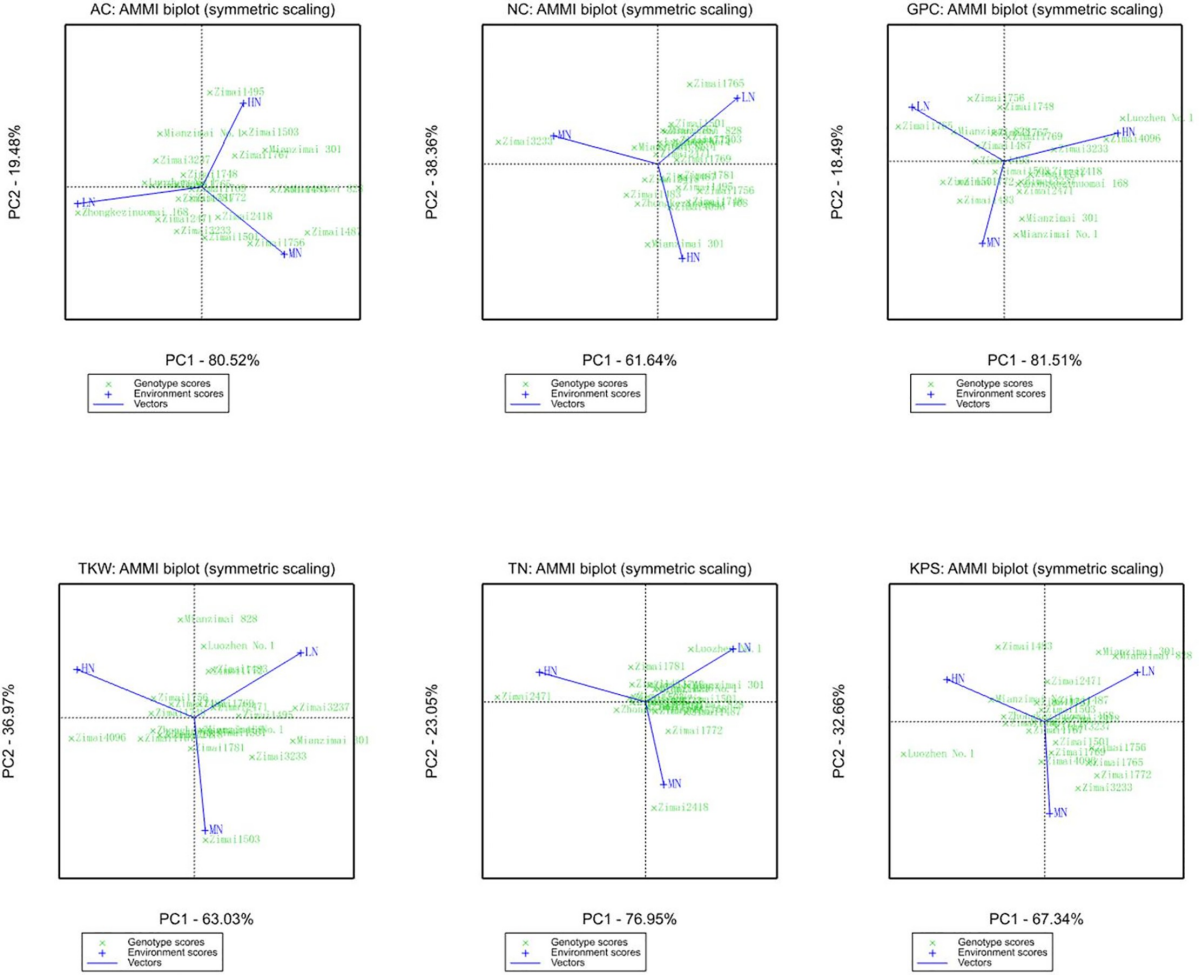
AMMI analysis of purple wheat lines for nutrient quality and yield composition traits.

For AC in purple genotypes (Fig 3, Table 6), ZKZNM168, Mianzimai 828 and Zimai1769 had higher AC across all N levels. The lowest ASV was observed in Zimai1769, which indicated that its high AC could be stable across all N levels. The highest ASV belonged to ZKZNM168, followed by Zimai1487 and Mianzimai 828, indicating that they are unstable with respect to N treatment. According to Fig 3, ZKZNM168, showing the highest AC, was the best adapted to environmental LN stress. In combination with other quality- and yield-related traits, ZKZNM168 also showed relatively low ASVs and moderate measured values (Table 6, S7 Table, S2 Fig), indicating that ZKZNM168 might be the most suitable genotype of the purple genotypes for planting under LN stress due to its high AC and stable performance for other quality and yield traits.

**Table 6:** Average and AMMI stability value (ASV) of nutrient quality traits and yield composition traits for the purple wheat lines.

Genotypes	Nutrient quality traits						Yield composition traits					
	AC (μg/g)		NC (μg/g)		GPC (%)		TKW (g)		TN		KPS	
	Mean	ASV	Mean	ASV	Mean	ASV	Mean	ASV	Mean	ASV	Mean	ASV
Zhongkezinuomai 168	60.48	10.25	23.76	0.65	15.23	0.42	45.28	0.92	7.65	1.78	54.18	2.33
Luozhen No.1	29.61	4.72	23.48	0.27	15.36	3.35	52.59	0.94	6.29	2.94	67.73	8.08
Mianzimai 828	40.21	7.00	19.34	0.43	12.96	1.44	44.87	1.29	6.67	1.31	55.39	4.40
Mianzimai No.1	25.70	3.62	24.01	0.52	15.81	0.58	45.70	0.22	7.08	0.63	68.78	3.09
Mianzimai 301	18.75	5.27	22.83	1.03	14.87	0.62	44.52	2.17	5.04	2.60	62.87	3.61
Zimai1483	20.32	5.86	23.23	0.74	14.94	1.28	43.24	0.74	8.69	0.89	59.63	3.36
Zimai1487	24.96	8.75	22.63	0.19	14.65	0.77	44.31	0.53	6.75	2.52	54.74	0.79
Zimai1495	17.25	2.00	21.85	0.51	14.49	0.81	43.35	0.96	8.63	0.79	61.49	2.22
Zimai1501	23.28	1.04	21.20	0.57	14.31	1.73	43.59	0.43	6.49	2.28	55.06	0.84
Zimai1503	19.48	3.67	21.15	0.66	14.51	0.26	44.10	1.59	8.10	0.98	57.83	0.37
Zimai1748	24.45	1.56	23.05	0.78	15.95	0.37	45.52	0.94	7.10	0.38	56.01	0.60
Zimai1756	25.29	4.15	24.39	0.91	16.23	1.04	45.54	0.92	7.01	0.12	51.09	2.77
Zimai1765	24.75	2.08	23.38	1.19	15.37	2.99	51.22	0.79	7.54	0.62	55.59	2.75
Zimai1767	20.73	2.80	22.84	0.43	15.07	0.34	50.74	1.21	7.70	0.80	55.76	0.93
Zimai1769	38.69	0.85	22.81	0.38	15.01	0.19	50.34	0.21	7.23	0.18	45.82	0.93
Zimai1772	15.58	0.88	23.80	0.46	15.80	1.27	50.08	0.67	8.06	1.48	44.13	3.32
Zimai1781	18.92	1.94	23.42	0.48	15.52	0.75	48.21	0.40	7.83	1.11	58.51	0.69
Zimai2471	20.75	3.69	22.47	0.13	14.86	0.47	44.08	0.44	13.64	9.08	58.76	1.11
Zimai2418	21.66	1.46	22.82	0.33	14.95	1.23	44.89	0.61	9.48	2.02	57.80	1.17
Zimai3233	23.65	2.26	25.09	3.23	14.94	1.41	42.52	1.36	7.79	0.10	58.77	2.64
Zimai3237	21.81	3.83	22.91	0.23	15.25	0.49	44.22	2.20	7.75	0.75	55.03	0.67
Zimai4096	25.12	4.08	23.77	0.60	15.21	2.88	42.79	2.69	6.66	0.84	58.78	1.12

The highlighted color is deepening with the corresponding average values or ASVs increasing

*AC* anthocyanin content, *NC* nitrogen concentration, *GPC* protein content, *TKW* thousand-kernel weight, *KPS* kernels per spike, *TN* tiller number.

### Expression of key genes for anthocyanin synthesis in wheat

**Fig 4:**
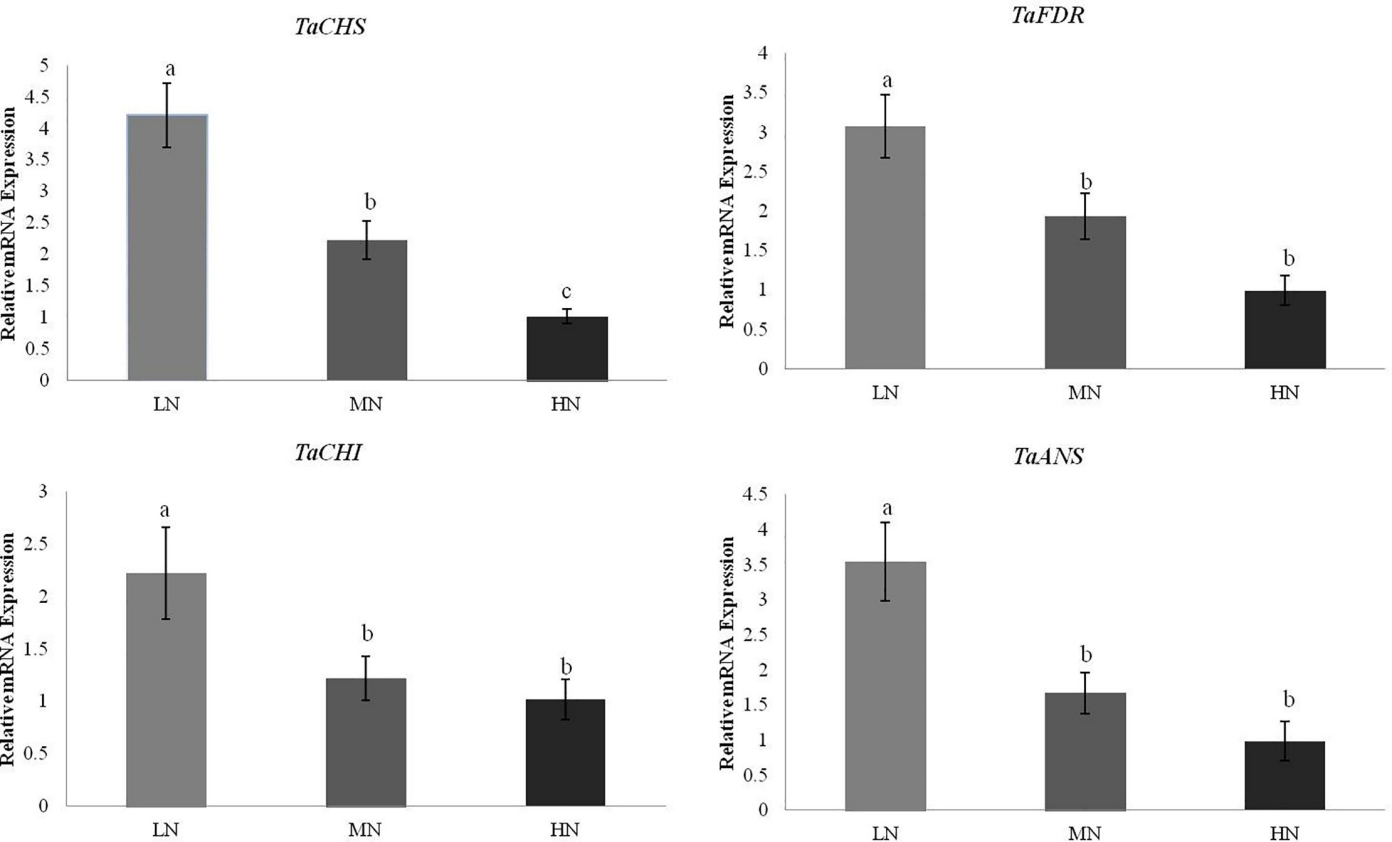
Expression of *TaCHS*, *TaFDR*, *TaCHI* and *TaANS* in Zhongkezinuomai168 (ZKZNM168). The different letters on the columns represent significant differences among LN, MN and HN at the level of 0.05.

To study the influence of nitrogen levels on AC in colored wheat, we detected the expression of four key genes involved in anthocyanin synthesis in ZKZNM168 (a purple wheat variety released by CIBCAS), by qRT-PCR. When tested at approximately 25 days after wheat pollination, the results showed that these four key genes (*TaCHS*, *TaFDR*, *TaCHI* and *TaANS*) were all downregulated when more nitrogen fertilizer was applied (Fig 4). This result was consistent with the previous result that AC in wheat decreased with increasing nitrogen fertilizer application.

## Discussion

Wheat is the most widely grown crop and a major source of protein for humans. Common-colored wheat grains are generally white or red, with very little color in the seed coat. With the improvement of living standards, an increasing pursuit of diversification and nutrition has been observed among breeders [26, 27, 28]. On the other hand, the benefits of planting wheat in China, especially in Southwest China, have not significantly increased in recent years, probably because of the increasing labor and land costs, which have caused fewer farmers to be willing to plant wheat on a large scale. Therefore, breeding highly nutritious wheat to increase its commercial value is an efficient method to improve wheat planting benefits. Blue and purple wheat are valuable germplasm resources with high levels of anthocyanins [12], which are responsible for oxidation resistance and might increase the nutritional value of wheat flour and processed foods [5, 15].

In previous studies, wheat quality traits, such as GPC, have been found to negatively correlate with yield-related traits because of the dilution effect [23, 29]. Hence, simultaneously improving quality and yield is always a difficult goal for wheat breeders. Anthocyanin is an unconventional but intriguing nutrient trait in wheat that differs significantly among varieties, highly correlated with grain color [30]. The relationship of AC with familiar quality and yield traits, such as GPC, WGC, MTR, TKW and KPS, deserves investigation, in order to facilitate to breed special usage color wheat varieties with satisfying yield. In this study, the correlation of a total of 20 grain quality- and yield-related traits with AC were investigated, including two nutrient quality traits, eight processing quality traits, three yield-related traits and seven grain morphology traits. The correlation analysis (Table 1, S4 and S5 Tables) demonstrated that AC was positively correlated with most processing quality traits, such as ZEL, WGC, FT and DST. In our opinion, the relation between AC and these processing quality traits might be indirectly controlled by its stable and remarkable positive correlation with NC (correlation coefficient: 0.617~0.681). N is a major element required for most biological compounds, such as protein, gluten and anthocyanins. NC had extremely positive relationships with GPC (0.823~0.947) and WGC (0.806~0.907) and further affected other related processing traits, such as ZEL (influenced by GPC), FT and DST (controlled by GPC and WGC) (Table 1, S4 and S5 Tables), in accordance with previous results [20, 21, 22, 23]. The three yield components TKW, TN and KPS are the most important contributors to yield formation. Among these three components, TKW is mainly controlled by genetic factors and has the highest heritability, so it is the most stable trait of these three [31]. It has been found to have a balancing effect between yield traits and quality traits [32]. In this study, AC also had a negative correlation with TKW, TN and KPS and showed an especially stable, significant correlation with TKW (Table 1, S4 and S5 Tables). Therefore, the relationship of AC with grain size and shape traits, as the major traits affecting TKW, was further investigated. The results indicated that smaller and longer grains might have higher AC, which would need further explanation at the molecular level.

Additionally, N is the crucial element affecting AC and the other quality, besides genetic factors [20, 21, 22, 23][23, 24]. Thus, to high efficiently plant speicial usage color wheat, the optimized N managements could be used to fine tune these processing traits. However, the N pollution caused by excessive N fertilizer application has pushed breeders to select highly N-efficient varieties with optimal tolerance to LN management [33]. Therefore, in consideration of the higher nutrient and commercial value of colored wheat, breeding colored wheat varieties well adapted to LN stress might have both economic and ecological importance.

Previous studies have demonstrated that wheat quality traits such as GPC, ZEL, WGC, GH, FT and DST are affected by N application [20, 21, 22, 23]. In this study, the results were consistent with those of these previous studies (Table 2), that is, most quality traits increased with more N fertilizer application. For yield-related traits, TN and KPS also increased when provided with a sufficient N supply, while TKW and its related morphological traits decreased, possibly due to a tradeoff with TN and KPS.

A previous study found that restricted N fertilization can lead to increased coloration and antioxidant capacity of colored wheat by shifting the plant metabolism towards increased anthocyanin synthesis. Moreover, excessive N fertilization enhances anthocyanin degradation. Some studies have also reported that increasing N supply decreases both anthocyanin synthesis and chlorophyll degradation but accelerates starch degradation in fruit [34]. The anthocyanin biosynthesis pathway includes phenylalanine ammonia lyase (PAL), 4-coumarate–CoA ligase (4CL), chalcone synthase (CHS), chalcone isomerase (CHI), flavanone 3-hydroxylase (F3H), flavonoid 3’-hydroxylase (F3’H), flavonoid 3’5’-hydroxylase (F3’5’H), dihydroflavonol 4-reductase (DFR), and anthocyanidin synthase/leucoanthocyanidin dioxygenase (ANS/LDOX). Among them, ANS catalyzes the conversion of leucoanthocyanidin into anthocyanidin, which requires glycosylation, methylation, and acylation to form stable anthocyanin. These modifications are mediated by glucosyltransferase, methyltransferase and acyltransferase [35]. These enzymes are encoded by the corresponding upstream, early, late, and modification genes. The upstream structural genes include *PAL* and *4CL*; the early structural genes include *CHS*, *CHI*, *F3H*, *F3’H* and *F3’5’H*; the late structural genes include *DFR* and *ANS*; and the modification genes include *GT*, *MT*, and *AT*. The regulatory genes for plant anthocyanin biosynthesis include genes encoding *MYB*, *bHLH* and *WD40* transcription factors, which control anthocyanin accumulation in the tissues by regulating the expression of structural genes [36, 37]. The expression of *CHS* and *DFR* in purple wheat showed tissue and cultivar specificity, and *DFR* had 3 copies; *CHS* in different cultivars contained a single nucleotide polymorphism [38, 39]. The results of this study, together with those of other recent publications (Fig 4) [40, 41], indicate that anthocyanins are produced in a highly regulated, fine-tuned, and nutrient-specific manner as a result of metabolic adaptation to nutrient stress. In particular, anthocyanin-specific genes, namely, *TaCHS*, *TaFDR*, *TaCHI* and *TaANS*, which are involved in anthocyanin glycosylation and sequestration in the vacuole, are highly expressed in response to nutrient deficiency (Fig 4), indicating that the observed anthocyanin accumulation is not simply the result of an upregulation of the general phenylpropanoid pathway, which would also yield other secondary metabolites, such as flavones [15]. Additionally, anthocyanin-rich fractions may be obtained from appropriate procedures prior to milling [42]. These fractions are also rich in fiber and minerals and could represent suitable ingredients for the production of staple foods such as pasta or bread. Processing whole grains to bake products such as bread, cookies or muffins improves phenolic acid bioaccessibility and bioavailability by releasing some of the insoluble bound phenolic acids and increasing the level of soluble or free phenolic acids [43].

Similarly, we found that AC could accumulate when N application was reduced in blue, purple and common-colored wheat (Table 3). In particular, the AC in purple wheat increased more when the plants were under LN stress (Table 4), but the yield and quality traits were negatively affected. Thus, overall, colored wheat is well adapted to LN stress for anthocyanin biosynthesis but better adapted to MN conditions for the purposes of yield or other quality characteristics.

To further identify colored wheat varieties suitable for LN planting, the AMMI model was used in this study. Finally, the wheat genotypes Lanmai2999 and ZKZNM168 were found to be the most suitable for LN stress, with the highest AC values and good performance in other quality and yield features (Tables 5 and 6, S6 and S7 Tables). However, the yield related traits need further improvement. On the other hand, if regardless of maximizing the AC, this study also could provide some information to select specialized N management for each wheat line to fine tune its producing or processing features. For example, Lanlimai-3 has the highest ZEL but sensitive to N levels (S6 Table). From S1 Fig, it is could deducible that moderate N can be enough for its higher ZEL; and Mianzimai No.1 has the highest hardness (S7 Table) and was adapted for use in low-input management systems to get higher GH (S2 Fig).

## Conclusion

This study presented a detailed investigation of the quality and yield-related traits of colored wheat at different N levels to provide a basis for breeding colored wheat with high tolerance to LN stress. Overall, blue wheat had the highest AC across all N levels, while purple wheat accumulated greater AC under LN stress. Additionally, although AC was found to increase when N application was reduced, some other related quality and yield traits were decreased. Thus, the colored wheat is well adapted to LN stress for anthocyanin accumalation but better adapted to MN conditions for the purposes of moderate yield or other quality characteristics. Two varieties each of blue wheat and purple wheat were identified as having the highest comprehensive performance when considering their AC values and adaptation to LN stress, as well as their maintenance of other important characteristics.

## Supporting information

S1 Table Pedigrees related to genotypes. (DOCX)

S2 Table Soil nitrogen contents of different nitrogen levels before sowing during 2016–2017 and 2017–2018. (DOCX)

S3 Table The primers used in this study. (DOCX)

S4 Table Phenotypic correlations among the 21 investigated traits under medium nitrogen level. (DOCX)

S5 Table Phenotypic correlations among the 21 investigated traits under high nitrogen level. (DOCX)

S6 Table Average and AMMI stability value (ASV) of processing quality traits and grain morphology traits for the blue wheat lines. (DOCX)

S7 Table Average and AMMI stability value (ASV) of processing quality traits and grain morphology traits for the blue wheat lines. (DOCX)

S1 Fig AMMI analysis of blue wheat lines for processing quality traits and grain morphology traits. (DOCX)

S2 Fig AMMI analysis of purple wheat lines for processing quality traits and grain morphology traits. (DOCX)
