# Supplementary material for: Identification of colored wheat genotypes with suitable quality and yield traits in response to low nitrogen input
Source: PLoS One. 2020 Apr 21;15(4):e0229535. doi: 10.1371/journal.pone.0229535 (PMC7173872; doi:10.1371/journal.pone.0229535)
Supplement: S1 Table — (DOCX) [file pone.0229535.s001.docx]

Table S1. Pedigrees related to genotypes.

| Genotypes | Types | Grain color | Hybridized combination Parent |
| --- | --- | --- | --- |
| Chuanlanmai No.1 | Cultivar | Blue | Unknown |
| Lanmai2895 | Line | Blue | Zhongkemai138×Lanlimai |
| Lanmai2909 | Line | Blue | Neimai22938×Lanlimai |
| Lanmai2999 | Line | Blue | Chuanmai107×Lanlimai |
| Lanmai3471 | Line | Blue | Chuanyu20×Lanlimai |
| Lanmai3624 | Line | Blue | Zhongkemai47×Lanlimai |
| Lanmai3707 | Line | Blue | Mianmai28×Lanlimai |
| Lanlimai | Cultivar | Blue | Unknown |
| Lanlimai-2 | Cultivar | Blue | Unknown |
| Lanlimai-3 | Cultivar | Blue | Unknown |
| Zhongkezinuomai 168 | Variety | Purple | Zhongkemai 138/Luozhen No.1 |
| Luozhen No.1 | Cultivar | Purple | Unknown |
| Mianzimai 828 | Variety | Purple | Unknown |
| Mianzimai No.1 | Variety | Purple | Unknown |
| Mianzimai 301 | Variety | Purple | Unknown |
| Zimai1483 | Line | Purple | Zhongkemai138/Zhongkezinuomai 168/R7 |
| Zimai1487 | Line | Purple | Zhongkemai138/Zhongkezinuomai 168//Zhongkemai138 |
| Zimai1495 | Line | Purple | Zhongkemai138/Zhongkezinuomai 168 |
| Zimai1501 | Line | Purple | Zhongkemai138/Zhongkezinuomai 168//Chuanyu18 |
| Zimai1503 | Line | Purple | Zhongkemai47/Zhongkezinuomai 168//Chuanyu18 |
| Zimai1748 | Line | Purple | Chuanyu16/Yr5//Zhongkezinuomai 168 |
| Zimai1756 | Line | Purple | Zhongkemai138/Zhongkezinuomai 168/98w958 |
| Zimai1765 | Line | Purple | Zhongkemai138/Zhongkezinuomai 168//Zhongkezinuomai 168 |
| Zimai1767 | Line | Purple | Zhongkemai138/Zhongkezinuomai 168//Chuanyu18 |
| Zimai1769 | Line | Purple | Zhongkezinuomai 168/Chuannong23 |
| Zimai1772 | Line | Purple | Chuannong23/Zhongkezinuomai 168//Chuannong23 |
| Zimai1781 | Line | Purple | Zhongkemai138/Zhongkezinuomai 168//R7 |
| Zimai2471 | Line | Purple | Zhongkemai138/Luozhen1// Zhongkezinuomai 168 |
| Zimai2418 | Line | Purple | Zhongkemai138/Zhongkezinuomai 168//13J159R |
| Zimai3233 | Line | Purple | Zhongkemai138/Zhongkezinuomai 168//Chuanyu16 |
| Zimai3237 | Line | Purple | Zhongkemai138/Zhongkezinuomai 168//Chuanmai42 |
| Zimai4096 | Line | Purple | Zhongkemai138/Zhongkezinuomai 168//G266 |
| Mianmai 45 | Variety | Common color | 07146-12-1/Guinong19-4 |
| Mianmai 38 | Variety | Common color | 07146-12-1/Guinong19-4 |
| Zhongkemai 138 | Variety | Common color | Chuanmai 42/Chuanyu 16 |
| Chuanyu 16 | Variety | Common color | 30020/8619-10 //Jinmai 30 |
| Chuanmai 42 | Variety | Common color | SynCD768/SW3243//Chuan6415 |
| Mianyang 33 | Variety | Common color | 1294/Mianyang86-5 |
| Mianyang 28 | Variety | Common color | T9350-1-4/Mianyng 11 |
