# Supplementary material for: Identification of colored wheat genotypes with suitable quality and yield traits in response to low nitrogen input
Source: PLoS One. 2020 Apr 21;15(4):e0229535. doi: 10.1371/journal.pone.0229535 (PMC7173872; doi:10.1371/journal.pone.0229535)
Supplement: S2 Table — (DOCX) [file pone.0229535.s002.docx]

Table S2. Soil nitrogen contents of different nitrogen levels before sowing during 2016-2017 and 2017-2018.

| Nitrogen levels | 2016-2017 | | | 2017-2018 | | |
| --- | --- | --- | --- | --- | --- | --- |
|  | NO^3^-N （mg/kg） | NH_4_-N（mg/kg） | N% | NO^3^-N（mg/kg） | NH_4_-N（mg/kg） | N% |
| Low Nitrogen | 7.88 | 1.96 | 0.15 | 3.51 | 1.00 | 0.15 |
| Medium Nitrogen | 7.78 | 1.79 | 0.16 | 7.40 | 1.00 | 0.16 |
