# Supplementary material for: Identification of colored wheat genotypes with suitable quality and yield traits in response to low nitrogen input
Source: PLoS One. 2020 Apr 21;15(4):e0229535. doi: 10.1371/journal.pone.0229535 (PMC7173872; doi:10.1371/journal.pone.0229535)
Supplement: S3 Table — (DOCX) [file pone.0229535.s003.docx]

Table S3. The primers used in this study.

| Primers | Sequences (3'-5') |
| --- | --- |
| *TaGAPDH-F* | AAGGCTGTTGGCAAGGTG |
| *TaGAPDH-R* | GTGGTCGTTCAGAGCAATCC |
| *TaCHS-F* | GAGGATGTGTGACAAATCGCAG |
| *TaCHS-R* | CTGCTGATACATCATGAGGCG |
| *TaFDR-F* | CACCGGCGTCTTCCACGTCGC |
| *TaFDR-R* | GACACGAAGTACACCATCCTG |
| *TaCHI-F* | GCAGTACTCGGACAAGGTGA |
| *TaCHI-R* | GTTCGTTCACACCGAAACC |
| *TaANS-F* | AGCTCAAGATCAACTACTAC |
| *TaANS-R* | GAAGACGACCCAGGAGA |
