# Supplementary material for: Identification of colored wheat genotypes with suitable quality and yield traits in response to low nitrogen input
Source: PLoS One. 2020 Apr 21;15(4):e0229535. doi: 10.1371/journal.pone.0229535 (PMC7173872; doi:10.1371/journal.pone.0229535)
Supplement: S4 Table — (DOCX) [file pone.0229535.s004.docx]

Table S4. Phenotypic correlations among the 21 investigated traits under medium nitrogen level.

|  | AC | NC | GPC | WGC | ZEL | GH | TW | WA | FT | DST | MTR | GA | GP | LWR | GL | GW | GD | GR | TKW | TN | KPS |
| --- | --- | --- | --- | --- | --- | --- | --- | --- | --- | --- | --- | --- | --- | --- | --- | --- | --- | --- | --- | --- | --- |
| AC | 1.000 |  |  |  |  |  |  |  |  |  |  |  |  |  |  |  |  |  |  |  |  |
| NC | 0.693 | 1.000 |  |  |  |  |  |  |  |  |  |  |  |  |  |  |  |  |  |  |  |
| GPC | 0.561 | 0.878 | 1.000 |  |  |  |  |  |  |  |  |  |  |  |  |  |  |  |  |  |  |
| WGC | 0.521 | 0.902 | 0.975 | 1.000 |  |  |  |  |  |  |  |  |  |  |  |  |  |  |  |  |  |
| ZEL | 0.583 | 0.847 | 0.924 | 0.912 | 1.000 |  |  |  |  |  |  |  |  |  |  |  |  |  |  |  |  |
| GH | 0.077 | 0.246 | 0.040 | 0.213 | 0.273 | 1.000 |  |  |  |  |  |  |  |  |  |  |  |  |  |  |  |
| TW | 0.455 | 0.378 | 0.220 | 0.174 | 0.394 | 0.130 | 1.000 |  |  |  |  |  |  |  |  |  |  |  |  |  |  |
| WA | 0.118 | 0.267 | 0.015 | 0.170 | 0.239 | 0.877 | 0.402 | 1.000 |  |  |  |  |  |  |  |  |  |  |  |  |  |
| FT | 0.500 | 0.794 | 0.656 | 0.595 | 0.758 | 0.503 | 0.651 | 0.589 | 1.000 |  |  |  |  |  |  |  |  |  |  |  |  |
| DST | 0.614 | 0.845 | 0.876 | 0.802 | 0.959 | 0.196 | 0.598 | 0.258 | 0.843 | 1.000 |  |  |  |  |  |  |  |  |  |  |  |
| MTR | 0.074 | 0.058 | -0.174 | -0.126 | -0.076 | 0.206 | 0.506 | 0.349 | 0.417 | 0.123 | 1.000 |  |  |  |  |  |  |  |  |  |  |
| GA | -0.297 | -0.151 | -0.228 | -0.119 | -0.237 | 0.288 | -0.119 | 0.287 | 0.040 | -0.240 | 0.062 | 1.000 |  |  |  |  |  |  |  |  |  |
| GP | 0.218 | 0.297 | 0.081 | 0.181 | 0.119 | 0.409 | 0.334 | 0.532 | 0.478 | 0.195 | 0.322 | 0.721 | 1.000 |  |  |  |  |  |  |  |  |
| LWR | 0.657 | 0.605 | 0.382 | 0.401 | 0.440 | 0.250 | 0.601 | 0.415 | 0.633 | 0.550 | 0.378 | -0.103 | 0.613 | 1.000 |  |  |  |  |  |  |  |
| GL | 0.429 | 0.464 | 0.224 | 0.300 | 0.276 | 0.392 | 0.487 | 0.540 | 0.600 | 0.373 | 0.381 | 0.449 | 0.941 | 0.841 | 1.000 |  |  |  |  |  |  |
| GW | -0.679 | -0.568 | -0.439 | -0.395 | -0.481 | -0.025 | -0.531 | -0.147 | -0.486 | -0.572 | -0.264 | 0.629 | -0.078 | -0.833 | -0.405 | 1.000 |  |  |  |  |  |
| GD | -0.296 | -0.140 | -0.212 | -0.103 | -0.225 | 0.289 | -0.114 | 0.289 | 0.054 | -0.227 | 0.067 | 0.999 | 0.722 | -0.101 | 0.451 | 0.628 | 1.000 |  |  |  |  |
| GR | -0.647 | -0.608 | -0.388 | -0.405 | -0.438 | -0.227 | -0.608 | -0.387 | -0.644 | -0.555 | -0.382 | 0.087 | -0.621 | -0.994 | -0.846 | 0.825 | 0.084 | 1.000 |  |  |  |
| TKW | -0.531 | -0.336 | -0.365 | -0.191 | -0.345 | 0.227 | -0.281 | 0.179 | -0.078 | -0.368 | 0.008 | 0.916 | 0.457 | -0.389 | 0.153 | 0.802 | 0.920 | 0.362 | 1.000 |  |  |
| TN | -0.315 | -0.202 | -0.177 | -0.191 | -0.209 | -0.137 | -0.352 | -0.366 | -0.296 | -0.281 | -0.241 | -0.090 | -0.373 | -0.412 | -0.430 | 0.251 | -0.100 | 0.390 | 0.051 | 1.000 |  |
| KPS | -0.004 | -0.126 | -0.154 | -0.090 | -0.211 | 0.155 | -0.126 | 0.105 | -0.088 | -0.245 | 0.098 | 0.102 | 0.076 | 0.007 | 0.059 | 0.055 | 0.099 | -0.002 | 0.080 | -0.275 | 1.000 |

Notes: The numbers which were highlighted by blue color indicates significance at the level of 0.05.

The numbers which were highlighted by purple color indicates significance at the level of 0.01.

*AC* anthocyanin content, *NC* nitrogen concentration, *GPC* protein content, *WGC* wet gluten content, *ZEL* Zeleny sedimentation value, *GH* grain hardness, *TW* test weight, *WA* water absorption, *FT* formation time, *DST* dough stabilization time, *MTR* maximum tensile resistance, *GL* grain length, *GW* grain width, *LWR* grain length/width ratio, *GD* grain diameter, *GA* grain area, *GP* grain perimeter, *GR* grain roundness, *TKW* thousand-kernel weight, *KPS* kernels per spike, *TN* tiller number.
