# Supplementary material for: Identification of colored wheat genotypes with suitable quality and yield traits in response to low nitrogen input
Source: PLoS One. 2020 Apr 21;15(4):e0229535. doi: 10.1371/journal.pone.0229535 (PMC7173872; doi:10.1371/journal.pone.0229535)
Supplement: S5 Table — (DOCX) [file pone.0229535.s005.docx]

Table S5. Phenotypic correlations among the 21 investigated traits under high nitrogen level.

|  | AC | NC | GPC | WGC | ZEL | GH | TW | WA | FT | DST | MTR | GA | GP | LWR | GL | GW | GD | GR | TKW | TN | KPS |
| --- | --- | --- | --- | --- | --- | --- | --- | --- | --- | --- | --- | --- | --- | --- | --- | --- | --- | --- | --- | --- | --- |
| AC | 1.000 |  |  |  |  |  |  |  |  |  |  |  |  |  |  |  |  |  |  |  |  |
| NC | 0.617 | 1.000 |  |  |  |  |  |  |  |  |  |  |  |  |  |  |  |  |  |  |  |
| GPC | 0.567 | 0.823 | 1.000 |  |  |  |  |  |  |  |  |  |  |  |  |  |  |  |  |  |  |
| WGC | 0.523 | 0.806 | 0.969 | 1.000 |  |  |  |  |  |  |  |  |  |  |  |  |  |  |  |  |  |
| ZEL | 0.556 | 0.702 | 0.890 | 0.846 | 1.000 |  |  |  |  |  |  |  |  |  |  |  |  |  |  |  |  |
| GH | -0.311 | -0.181 | -0.282 | -0.096 | -0.073 | 1.000 |  |  |  |  |  |  |  |  |  |  |  |  |  |  |  |
| TW | 0.480 | 0.317 | 0.336 | 0.322 | 0.548 | 0.196 | 1.000 |  |  |  |  |  |  |  |  |  |  |  |  |  |  |
| WA | -0.105 | -0.042 | -0.095 | 0.069 | 0.146 | 0.907 | 0.427 | 1.000 |  |  |  |  |  |  |  |  |  |  |  |  |  |
| FT | 0.320 | 0.544 | 0.620 | 0.733 | 0.654 | 0.336 | 0.666 | 0.487 | 1.000 |  |  |  |  |  |  |  |  |  |  |  |  |
| DST | 0.571 | 0.663 | 0.852 | 0.804 | 0.971 | -0.120 | 0.659 | 0.141 | 0.722 | 1.000 |  |  |  |  |  |  |  |  |  |  |  |
| MTR | 0.270 | 0.057 | 0.193 | 0.205 | 0.453 | 0.249 | 0.632 | 0.426 | 0.588 | 0.566 | 1.000 |  |  |  |  |  |  |  |  |  |  |
| GA | -0.365 | -0.280 | -0.225 | -0.069 | -0.313 | 0.482 | -0.192 | 0.359 | 0.093 | -0.334 | -0.275 | 1.000 |  |  |  |  |  |  |  |  |  |
| GP | 0.115 | 0.116 | 0.140 | 0.283 | 0.108 | 0.499 | 0.269 | 0.580 | 0.521 | 0.137 | 0.161 | 0.714 | 1.000 |  |  |  |  |  |  |  |  |
| LWR | 0.618 | 0.519 | 0.487 | 0.491 | 0.541 | 0.074 | 0.599 | 0.336 | 0.607 | 0.607 | 0.550 | -0.245 | 0.501 | 1.000 |  |  |  |  |  |  |  |
| GL | 0.353 | 0.303 | 0.308 | 0.411 | 0.309 | 0.390 | 0.453 | 0.556 | 0.630 | 0.357 | 0.346 | 0.411 | 0.930 | 0.779 | 1.000 |  |  |  |  |  |  |
| GW | -0.642 | -0.531 | -0.473 | -0.389 | -0.560 | 0.219 | -0.549 | -0.036 | -0.395 | -0.625 | -0.541 | 0.723 | 0.037 | -0.844 | -0.327 | 1.000 |  |  |  |  |  |
| GD | -0.370 | -0.283 | -0.225 | -0.069 | -0.316 | 0.477 | -0.200 | 0.352 | 0.091 | -0.337 | -0.279 | 0.999 | 0.707 | -0.256 | 0.402 | 0.731 | 1.000 |  |  |  |  |
| GR | -0.618 | -0.510 | -0.476 | -0.485 | -0.534 | -0.086 | -0.641 | -0.359 | -0.633 | -0.612 | -0.566 | 0.227 | -0.512 | -0.992 | -0.786 | 0.832 | 0.239 | 1.000 |  |  |  |
| TKW | -0.518 | -0.471 | -0.424 | -0.296 | -0.461 | 0.440 | -0.273 | 0.261 | -0.119 | -0.484 | -0.323 | 0.913 | 0.432 | -0.537 | 0.088 | 0.883 | 0.917 | 0.508 | 1.000 |  |  |
| TN | -0.386 | -0.261 | -0.246 | -0.272 | -0.341 | -0.227 | -0.427 | -0.438 | -0.362 | -0.370 | -0.419 | 0.043 | -0.328 | -0.506 | -0.452 | 0.367 | 0.044 | 0.520 | 0.142 | 1.000 |  |
| KPS | -0.080 | -0.099 | -0.106 | -0.079 | -0.184 | 0.190 | -0.012 | 0.054 | -0.075 | -0.256 | -0.122 | 0.032 | -0.044 | -0.090 | -0.070 | 0.091 | 0.026 | 0.103 | -0.019 | 0.156 | 1.000 |

Notes: The numbers which were highlighted by blue color indicates significance at the level of 0.05.

The numbers which were highlighted by purple color indicates significance at the level of 0.01. *AC* anthocyanin content, *NC* nitrogen concentration, *GPC* protein content, *WGC* wet gluten content, *ZEL* Zeleny sedimentation value, *GH* grain hardness, *TW* test weight, *WA* water absorption, *FT* formation time, *DST* dough stabilization time, *MTR* maximum tensile resistance, *GL* grain length, *GW* grain width, *LWR* grain length/width ratio, *GD* grain diameter, *GA* grain area, *GP* grain perimeter, *GR* grain roundness, *TKW* thousand-kernel weight, *KPS* kernels per spike, *TN* tiller number.
