# Supplementary material for: Identification of colored wheat genotypes with suitable quality and yield traits in response to low nitrogen input
Source: PLoS One. 2020 Apr 21;15(4):e0229535. doi: 10.1371/journal.pone.0229535 (PMC7173872; doi:10.1371/journal.pone.0229535)
Supplement: S6 Table — (DOCX) [file pone.0229535.s006.docx]

Table S6. Average and AMMI stability value (ASV) of processing quality traits and grain morphology traits for the blue wheat lines.

| Genotypes | Processing quality traits | | | | | | | | | | | | | | | | Grain morphology traits | | | | | | | | | | | | | |
| --- | --- | --- | --- | --- | --- | --- | --- | --- | --- | --- | --- | --- | --- | --- | --- | --- | --- | --- | --- | --- | --- | --- | --- | --- | --- | --- | --- | --- | --- | --- |
|  | WGC(%) | | ZEL(ml) | | GH | | TW(g/L) | | WA(%) | | FT(min) | | DST(min) | | MTR(B.U.) | | GA(mm^2^) | | GP(mm) | | LWR | | GL(mm) | | GW(mm) | | GD(mm) | | GR | |
|  | Mean | ASV | Mean | ASV | Mean | ASV | Mean | ASV | Mean | ASV | Mean | ASV | Mean | ASV | Mean | ASV | Mean | ASV | Mean | ASV | Mean | ASV | Mean | ASV | Mean | ASV | Mean | ASV | Mean | ASV |
| Chuanlanmai No.1 | 30.67 | 0.72 | 38.27 | 2.12 | 63.46 | 2.50 | 770.50 | 2.12 | 61.82 | 3.19 | 2.72 | 1.64 | 11.42 | 2.16 | 644.20 | 5.00 | 16.33 | 0.29 | 16.68 | 0.34 | 2.18 | 0.26 | 6.67 | 0.19 | 3.10 | 0.22 | 4.53 | 0.15 | 0.47 | 0.11 |
| Lanmai2895 | 34.45 | 1.65 | 48.11 | 2.01 | 68.74 | 2.90 | 782.80 | 1.73 | 64.71 | 3.59 | 3.59 | 0.28 | 17.10 | 1.99 | 564.20 | 10.45 | 17.87 | 0.47 | 17.76 | 0.28 | 2.33 | 0.21 | 7.22 | 0.21 | 3.12 | 0.28 | 4.74 | 0.18 | 0.44 | 0.11 |
| Lanmai2909 | 33.95 | 0.27 | 44.98 | 1.01 | 64.16 | 0.96 | 766.70 | 3.15 | 60.87 | 1.25 | 2.98 | 0.39 | 14.81 | 1.32 | 674.30 | 3.70 | 17.84 | 0.65 | 17.86 | 0.43 | 2.35 | 0.22 | 7.30 | 0.26 | 3.13 | 0.27 | 4.74 | 0.26 | 0.43 | 0.06 |
| Lanmai2999 | 33.17 | 0.66 | 43.20 | 1.34 | 44.43 | 0.80 | 773.60 | 3.70 | 49.74 | 3.10 | 2.16 | 0.42 | 15.02 | 1.13 | 605.50 | 3.19 | 13.54 | 0.46 | 15.39 | 0.53 | 2.32 | 0.26 | 6.24 | 0.25 | 2.73 | 0.15 | 4.12 | 0.18 | 0.44 | 0.09 |
| Lanmai3471 | 36.18 | 2.61 | 52.83 | 2.13 | 63.21 | 0.63 | 773.50 | 1.08 | 64.15 | 1.83 | 3.77 | 1.68 | 20.90 | 1.45 | 686.10 | 9.88 | 15.55 | 0.62 | 16.70 | 0.44 | 2.40 | 0.42 | 6.84 | 0.14 | 2.89 | 0.30 | 4.42 | 0.24 | 0.43 | 0.13 |
| Lanmai3624 | 32.33 | 1.25 | 42.05 | 0.98 | 69.50 | 1.01 | 763.30 | 0.74 | 62.44 | 0.80 | 3.25 | 1.20 | 12.67 | 1.00 | 743.70 | 3.37 | 16.49 | 0.55 | 16.48 | 0.19 | 2.08 | 1.43 | 6.52 | 0.24 | 3.17 | 0.57 | 4.55 | 0.22 | 0.49 | 0.56 |
| Lanmai3707 | 33.68 | 0.78 | 47.68 | 1.50 | 66.72 | 1.61 | 767.80 | 4.69 | 63.16 | 2.23 | 3.21 | 1.88 | 16.32 | 1.63 | 726.10 | 10.14 | 15.48 | 1.16 | 17.09 | 0.36 | 2.59 | 1.62 | 7.09 | 0.22 | 2.78 | 0.81 | 4.40 | 0.45 | 0.40 | 0.49 |
| Lanlimai | 36.81 | 1.10 | 52.76 | 0.82 | 61.72 | 0.33 | 783.90 | 1.66 | 62.60 | 1.67 | 4.07 | 0.14 | 21.61 | 0.55 | 621.30 | 3.31 | 15.17 | 0.70 | 16.42 | 0.80 | 2.35 | 0.17 | 6.67 | 0.41 | 2.86 | 0.23 | 4.37 | 0.27 | 0.43 | 0.04 |
| Lanlimai-2 | 32.66 | 1.54 | 44.88 | 1.76 | 63.74 | 1.81 | 769.20 | 3.71 | 61.54 | 4.72 | 2.52 | 2.01 | 14.62 | 1.44 | 542.40 | 4.33 | 15.63 | 0.71 | 16.88 | 0.38 | 2.50 | 0.53 | 7.00 | 0.14 | 2.84 | 0.34 | 4.43 | 0.25 | 0.41 | 0.15 |
| Lanlimai-3 | 34.18 | 1.76 | 53.72 | 3.14 | 64.84 | 1.13 | 796.60 | 2.30 | 63.45 | 0.73 | 3.86 | 2.74 | 22.49 | 3.68 | 798.80 | 12.34 | 15.55 | 0.59 | 16.58 | 0.59 | 2.32 | 0.68 | 6.73 | 0.36 | 2.94 | 0.43 | 4.42 | 0.27 | 0.43 | 0.30 |

*WGC* wet gluten content, *ZEL* Zeleny sedimentation value, *GH* grain hardness, *TW* test weight, *WA* water absorption, *FT* formation time, *DST* dough stabilization time, *MTR* maximum tensile resistance, *GL* grain length, *GW* grain width, *LWR* grain length/width ratio, *GD* grain diameter, *GA* grain area, *GP* grain perimeter, *GR* grain roundness.
