# Supplementary material for: Identification of colored wheat genotypes with suitable quality and yield traits in response to low nitrogen input
Source: PLoS One. 2020 Apr 21;15(4):e0229535. doi: 10.1371/journal.pone.0229535 (PMC7173872; doi:10.1371/journal.pone.0229535)
Supplement: S7 Table — (DOCX) [file pone.0229535.s007.docx]

Table S7. Average and AMMI stability value (ASV) of processing quality traits and grain morphology traits for the purple wheat lines.

| Genotypes | Processing quality traits | | | | | | | | | | | | | | | | Grain morphology traits | | | | | | | | | | | | | |
| --- | --- | --- | --- | --- | --- | --- | --- | --- | --- | --- | --- | --- | --- | --- | --- | --- | --- | --- | --- | --- | --- | --- | --- | --- | --- | --- | --- | --- | --- | --- |
|  | WGC(%) | | ZEL(ml) | | GH | | TW(g/L) | | WA(%) | | FT(min) | | DST(min) | | MTR(B.U.) | | GA(mm^2^) | | GP(mm) | | LWR | | GL(mm) | | GW(mm) | | GD(mm) | | GR | |
|  | Mean | ASV | Mean | ASV | Mean | ASV | Mean | ASV | Mean | ASV | Mean | ASV | Mean | ASV | Mean | ASV | Mean | ASV | Mean | ASV | Mean | ASV | Mean | ASV | Mean | ASV | Mean | ASV | Mean | ASV |
| Zhongkezinuomai 168 | 31.19 | 0.35 | 40.38 | 0.32 | 58.51 | 17.11 | 755.40 | 1.22 | 56.01 | 2.07 | 1.84 | 0.18 | 10.90 | 0.53 | 430.00 | 8.22 | 15.92 | 0.86 | 15.81 | 0.53 | 1.92 | 0.05 | 6.12 | 0.29 | 3.22 | 0.19 | 4.47 | 0.30 | 0.53 | 0.03 |
| Luozhen No.1 | 33.01 | 3.85 | 41.16 | 2.68 | 72.84 | 5.07 | 763.20 | 7.08 | 67.02 | 0.41 | 2.82 | 1.21 | 10.42 | 4.24 | 468.10 | 7.65 | 17.81 | 0.66 | 16.93 | 0.48 | 1.96 | 0.20 | 6.61 | 0.31 | 3.40 | 0.24 | 4.73 | 0.23 | 0.52 | 0.09 |
| Mianzimai 828 | 25.63 | 0.57 | 37.30 | 1.18 | 64.87 | 73.11 | 773.60 | 0.64 | 62.82 | 6.68 | 0.86 | 0.45 | 8.97 | 0.74 | 480.50 | 12.78 | 16.04 | 1.26 | 15.97 | 0.71 | 1.94 | 0.25 | 6.19 | 0.50 | 3.24 | 0.40 | 4.48 | 0.48 | 0.53 | 0.14 |
| Mianzimai No.1 | 33.13 | 1.05 | 47.69 | 0.86 | 70.67 | 18.11 | 777.80 | 4.33 | 65.95 | 2.51 | 3.15 | 0.88 | 15.76 | 0.76 | 582.20 | 6.24 | 16.40 | 0.21 | 16.15 | 0.38 | 1.95 | 0.47 | 6.25 | 0.49 | 3.27 | 0.13 | 4.53 | 0.07 | 0.53 | 0.23 |
| Mianzimai 301 | 30.32 | 2.13 | 42.56 | 1.77 | 64.83 | 45.59 | 777.00 | 3.68 | 62.44 | 4.29 | 2.29 | 0.52 | 13.27 | 1.33 | 653.50 | 5.58 | 15.80 | 1.31 | 16.30 | 0.64 | 2.15 | 0.35 | 6.52 | 0.33 | 3.08 | 0.44 | 4.45 | 0.45 | 0.48 | 0.15 |
| Zimai1483 | 30.45 | 1.64 | 39.13 | 1.03 | 57.52 | 7.18 | 754.60 | 0.03 | 55.26 | 1.18 | 1.68 | 0.27 | 10.26 | 0.70 | 479.10 | 2.65 | 15.52 | 0.29 | 15.57 | 0.17 | 1.91 | 0.29 | 6.02 | 0.18 | 3.19 | 0.16 | 4.41 | 0.11 | 0.54 | 0.13 |
| Zimai1487 | 29.81 | 1.37 | 37.16 | 1.35 | 57.37 | 8.25 | 753.60 | 1.14 | 54.71 | 2.80 | 1.43 | 0.30 | 8.81 | 1.08 | 476.10 | 11.01 | 15.73 | 0.16 | 15.66 | 0.05 | 1.90 | 0.12 | 6.05 | 0.06 | 3.22 | 0.15 | 4.44 | 0.06 | 0.54 | 0.08 |
| Zimai1495 | 29.36 | 1.23 | 38.14 | 0.49 | 58.79 | 7.12 | 755.50 | 1.22 | 55.74 | 0.28 | 1.52 | 0.47 | 9.75 | 0.46 | 539.70 | 1.45 | 15.28 | 0.56 | 15.49 | 0.19 | 1.92 | 0.08 | 6.01 | 0.04 | 3.17 | 0.17 | 4.38 | 0.19 | 0.53 | 0.05 |
| Zimai1501 | 29.02 | 2.68 | 37.42 | 1.30 | 58.47 | 4.80 | 755.40 | 1.93 | 55.64 | 0.65 | 1.37 | 0.47 | 9.17 | 1.37 | 511.10 | 10.35 | 15.45 | 0.29 | 15.58 | 0.03 | 1.93 | 0.12 | 6.05 | 0.05 | 3.18 | 0.12 | 4.41 | 0.09 | 0.53 | 0.05 |
| Zimai1503 | 29.58 | 0.95 | 38.38 | 0.89 | 58.80 | 13.68 | 756.00 | 0.60 | 55.46 | 1.52 | 1.67 | 0.53 | 10.10 | 1.25 | 521.40 | 10.79 | 15.63 | 0.69 | 15.63 | 0.47 | 1.91 | 0.16 | 6.04 | 0.31 | 3.21 | 0.26 | 4.43 | 0.27 | 0.54 | 0.07 |
| Zimai1748 | 32.66 | 0.58 | 42.47 | 0.94 | 55.06 | 7.73 | 755.50 | 1.45 | 55.00 | 1.42 | 2.10 | 0.33 | 12.93 | 0.84 | 472.50 | 3.16 | 16.06 | 0.59 | 15.88 | 0.36 | 1.91 | 0.25 | 6.13 | 0.27 | 3.23 | 0.22 | 4.50 | 0.21 | 0.53 | 0.11 |
| Zimai1756 | 33.26 | 1.50 | 43.98 | 0.56 | 56.06 | 0.93 | 757.20 | 0.60 | 55.67 | 0.88 | 2.33 | 0.43 | 13.98 | 0.61 | 512.80 | 4.80 | 15.90 | 0.62 | 15.74 | 0.24 | 1.88 | 0.11 | 6.06 | 0.09 | 3.24 | 0.17 | 4.47 | 0.19 | 0.54 | 0.06 |
| Zimai1765 | 31.57 | 4.46 | 39.19 | 1.71 | 58.55 | 3.90 | 753.90 | 0.75 | 56.50 | 0.19 | 1.97 | 0.78 | 10.44 | 1.62 | 490.80 | 2.38 | 17.54 | 0.64 | 16.76 | 0.32 | 1.97 | 0.05 | 6.53 | 0.16 | 3.35 | 0.13 | 4.70 | 0.23 | 0.52 | 0.01 |
| Zimai1767 | 30.89 | 0.63 | 38.42 | 0.55 | 58.03 | 9.02 | 754.10 | 0.62 | 56.15 | 0.99 | 1.78 | 0.24 | 9.94 | 0.36 | 453.00 | 6.27 | 17.53 | 0.83 | 16.72 | 0.38 | 1.96 | 0.19 | 6.51 | 0.20 | 3.35 | 0.32 | 4.69 | 0.32 | 0.52 | 0.11 |
| Zimai1769 | 30.75 | 0.29 | 36.77 | 0.40 | 54.52 | 3.54 | 750.90 | 0.60 | 55.61 | 1.13 | 1.29 | 0.10 | 8.76 | 0.49 | 400.20 | 6.51 | 17.40 | 0.33 | 16.53 | 0.16 | 1.90 | 0.02 | 6.35 | 0.06 | 3.39 | 0.09 | 4.68 | 0.12 | 0.54 | 0.01 |
| Zimai1772 | 32.61 | 1.82 | 40.64 | 0.84 | 56.52 | 3.53 | 753.30 | 1.08 | 55.62 | 1.10 | 2.00 | 0.43 | 11.28 | 0.89 | 464.60 | 3.66 | 17.01 | 0.22 | 16.55 | 0.19 | 1.98 | 0.07 | 6.49 | 0.12 | 3.31 | 0.09 | 4.63 | 0.09 | 0.52 | 0.01 |
| Zimai1781 | 32.05 | 1.16 | 40.81 | 0.37 | 57.95 | 9.38 | 755.90 | 1.54 | 56.45 | 0.42 | 2.32 | 0.17 | 12.17 | 0.35 | 527.10 | 1.94 | 16.65 | 0.20 | 16.25 | 0.11 | 1.95 | 0.06 | 6.33 | 0.08 | 3.27 | 0.09 | 4.58 | 0.07 | 0.52 | 0.03 |
| Zimai2471 | 30.41 | 0.62 | 38.22 | 0.18 | 57.09 | 12.75 | 754.90 | 1.30 | 54.58 | 1.89 | 1.76 | 0.07 | 9.81 | 0.36 | 474.00 | 5.05 | 15.68 | 0.15 | 15.68 | 0.07 | 1.92 | 0.17 | 6.06 | 0.14 | 3.20 | 0.07 | 4.44 | 0.05 | 0.53 | 0.08 |
| Zimai2418 | 30.53 | 1.49 | 39.02 | 0.87 | 58.03 | 16.70 | 756.80 | 1.27 | 55.23 | 1.00 | 1.82 | 0.21 | 10.21 | 1.04 | 470.70 | 4.07 | 15.76 | 0.43 | 15.71 | 0.34 | 1.92 | 0.18 | 6.07 | 0.26 | 3.21 | 0.14 | 4.44 | 0.17 | 0.53 | 0.09 |
| Zimai3233 | 30.25 | 1.92 | 39.02 | 0.31 | 57.58 | 18.23 | 758.60 | 1.76 | 54.55 | 1.69 | 1.68 | 0.14 | 10.19 | 0.36 | 423.70 | 4.88 | 15.06 | 0.62 | 15.35 | 0.23 | 1.91 | 0.10 | 5.95 | 0.09 | 3.14 | 0.24 | 4.35 | 0.23 | 0.54 | 0.06 |
| Zimai3237 | 31.38 | 0.93 | 40.83 | 0.60 | 58.51 | 7.97 | 751.80 | 0.28 | 56.44 | 1.08 | 1.91 | 0.14 | 11.35 | 0.76 | 482.80 | 15.47 | 15.79 | 1.19 | 15.77 | 0.63 | 1.92 | 0.17 | 6.10 | 0.30 | 3.20 | 0.29 | 4.45 | 0.46 | 0.53 | 0.07 |
| Zimai4096 | 31.87 | 3.82 | 39.17 | 2.87 | 65.76 | 4.49 | 744.10 | 1.51 | 61.72 | 1.99 | 1.84 | 0.98 | 9.31 | 2.61 | 551.00 | 12.41 | 15.44 | 0.88 | 15.34 | 0.48 | 1.81 | 0.10 | 5.83 | 0.25 | 3.26 | 0.24 | 4.40 | 0.36 | 0.57 | 0.06 |

*WGC* wet gluten content, *ZEL* Zeleny sedimentation value, *GH* grain hardness, *TW* test weight, *WA* water absorption, *FT* formation time, *DST* dough stabilization time, *MTR* maximum tensile resistance, *GL* grain length, *GW* grain width, *LWR* grain length/width ratio, *GD* grain diameter, *GA* grain area, *GP* grain perimeter, *GR* grain roundness.
