# Supplementary material for: Identification of colored wheat genotypes with suitable quality and yield traits in response to low nitrogen input
Source: PLoS One. 2020 Apr 21;15(4):e0229535. doi: 10.1371/journal.pone.0229535 (PMC7173872; doi:10.1371/journal.pone.0229535)
Supplement: S1 Fig — (DOCX) [file pone.0229535.s008.docx]

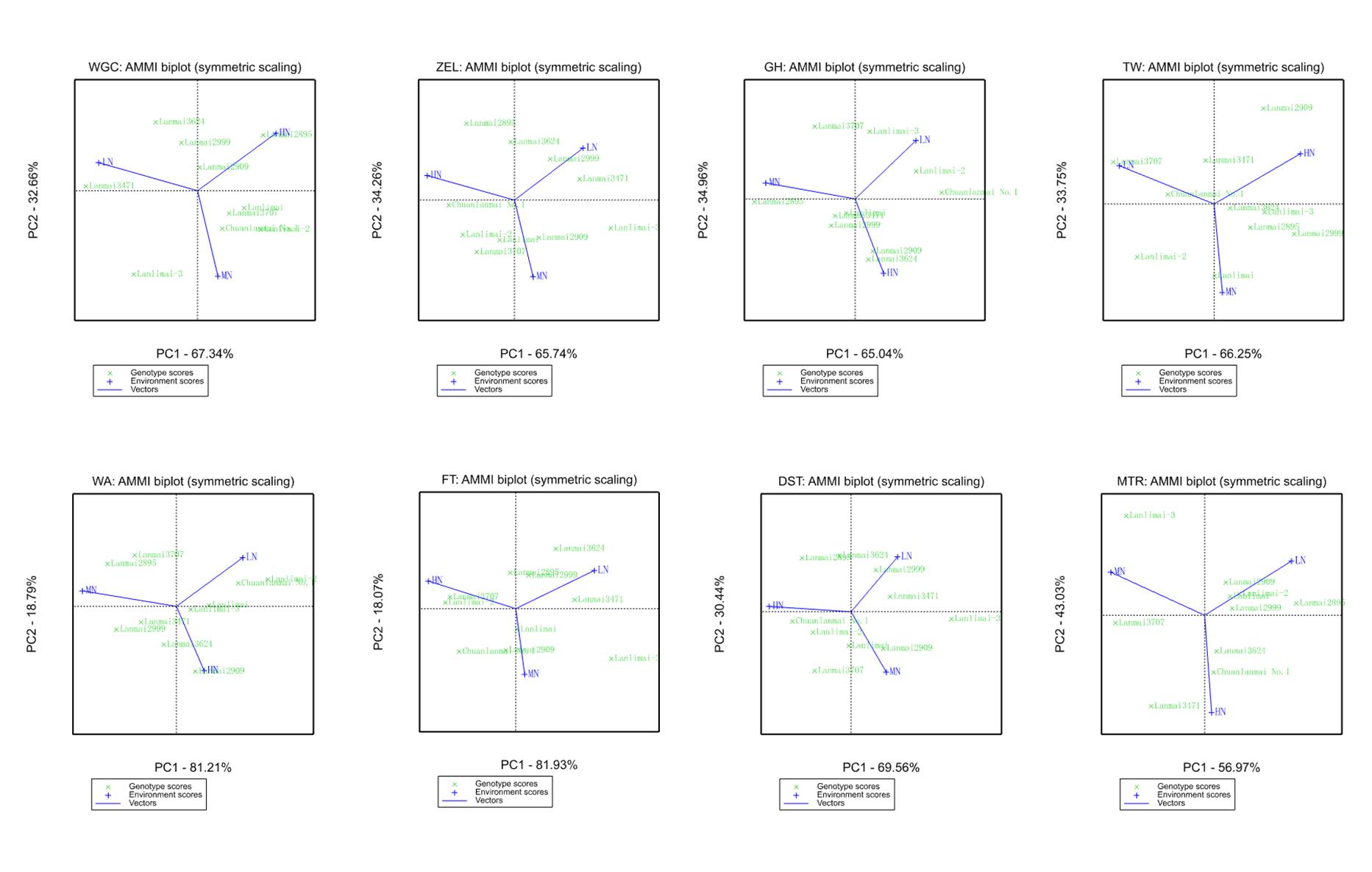

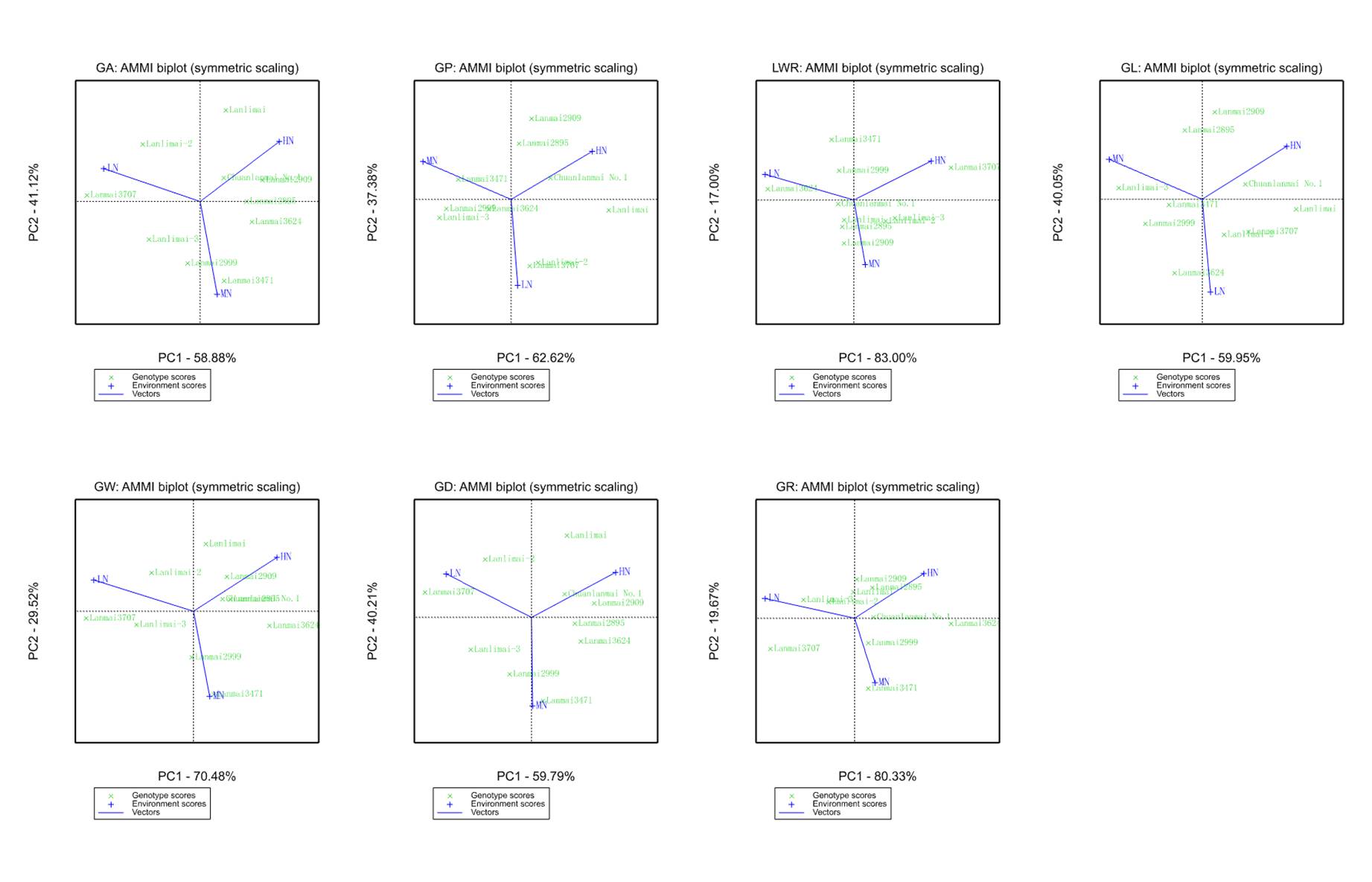


Fig. S1 AMMI analysis of blue wheat lines for processing quality traits and grain morphology traits.
